# Virus–pathogen interactions improve water quality along the Middle Route of the South-to-North Water Diversion Canal

**DOI:** 10.1038/s41396-023-01481-2

**Published:** 2023-07-31

**Authors:** Tianyi Chen, Tang Liu, Zongzhi Wu, Bingxue Wang, Qian Chen, Mi Zhang, Enhang Liang, Jinren Ni

**Affiliations:** 1 Eco-environment and Resource Efficiency Research Laboratory, School of Environment and Energy, Peking University Shenzhen Graduate School, Shenzhen, 518055 PR China; 2 Environmental Microbiome and Innovative Genomics Laboratory, College of Environmental Sciences and Engineering, Peking University, Beijing, 100871 PR China; 3 Environmental Microbiome Engineering and Innovative Genomics Laboratory, College of Chemistry and Environmental Engineering, Shenzhen University, Shenzhen, 518060 PR China; 4 State Environmental Protection Key Laboratory of All Materials Fluxes in River Ecosystems, Peking University, Beijing, 100871 PR China; 5 State Key Laboratory of Eco-hydraulics in Northwest Arid Region of China, Xi’an University of Technology, Xi’an, 710048 PR China

**Keywords:** Metagenomics, Metagenomics, Microbial ecology

## Abstract

Bacterial pathogens and viruses are the leading causes of global waterborne diseases. Here, we discovered an interesting natural paradigm of water “self-purification” through virus–pathogen interactions over a 1432 km continuum along the Middle Route of the South-to-North Water Diversion Canal (MR-SNWDC) in China, the largest water transfer project in the world. Due to the extremely low total phosphorus (TP) content (ND-0.02 mg/L) in the MR-SNWDC, the whole canal has experienced long-lasting phosphorus (P) limitation since its operation in 2015. Based on 4443 metagenome-assembled genomes (MAGs) and 40,261 nonredundant viral operational taxonomic units (vOTUs) derived from our recent monitoring campaign, we found that residential viruses experiencing extreme P constraints had to adopt special adaptive strategies by harboring smaller genomes to minimize nucleotide replication, DNA repair, and posttranslational modification costs. With the decreasing P supply downstream, bacterial pathogens showed repressed environmental fitness and growth potential, and a weakened capacity to maintain P acquisition, membrane formation, and ribonucleotide biosynthesis. Consequently, the unique viral predation effects under P limitation, characterized by enhanced viral lytic infections and an increased abundance of ribonucleotide reductase (RNR) genes linked to viral nuclear DNA replication cycles, led to unexpectedly lower health risks from waterborne bacterial pathogens in the downstream water-receiving areas. These findings highlighted the great potential of water self-purification associated with virus–pathogen dynamics for water-quality improvement and sustainable water resource management.

## Introduction

Bacterial pathogens are risk-spreading populations in varying environments, while viruses show the most diverse and frequent interplay as natural competitors and predators of these pathogens. Typical viral lifestyles include lysogenic, lytic, and chronic infection cycles [1], with virulent viruses contributing to immediate host lysis [2] and temperate viruses integrating genomes into host cells during the lysogenic period [3]. As viral replication and assembly rely heavily on hosts for nutrients and energy, frequent interactions between cells and viruses under nutrient-limited conditions, particularly in phosphorous (P)-constrained environments, might regulate pathogen population dynamics and consequently affect water quality in aquatic ecosystems.

Large repertoires of aquatic microbes exhibit sensitive dynamics associated with multiple environmental signals. Shifts in temperature, pH, and nutrient contents act as sources of natural selection for species with high adaptability and result in high-level fluctuation of community composition [4]. Nutrients such as P and nitrogen (N) are the primary growth-limiting factors of phototrophic microbes and could further affect the productivity of heterotrophs through the microbial loop [5]. In ecological theory, Liebig’s law of the minimum implies that the growth potential of microorganisms may depend on which nutrient is the most limiting [6]. Three benchmark values are provided for the N- or P-oligotrophic boundary based on the combinations of total N (TN) and total P (TP) concentration, including Dodd’s criteria (TP < 0.025 mg/L, TN < 0.7 mg/L), the UK’s water quality standard (TP < 0.02 mg/L, TN < 1.5 mg/L), and Norway’s water quality standard (TP < 0.02 mg/L, TN < 0.6 mg/L) [7, 8]. Nutrient fractions (especially the N:P ratio) also help define nutrition limitations in particular environments. For example, the Redfield ratio of N:P provides an “optimum” nutrient stoichiometric ratio (16:1) as a reference for marine and freshwater phytoplankton. Since a consensus on the ideal nutrient stoichiometric ratio has not been reached for bacteria, the Redfield ratio has been used approximately to infer potential N-limited or P-limited conditions [9]. Some P-limited ocean regions of wide concern displayed higher N:P ratios than the Redfield value according to the long-term time-series monitoring data, e.g., in the eastern Mediterranean (~28:1), the Bermuda Atlantic Time-series Study site (>24:1), and the station ALOHA in the North Pacific subtropical gyre (16:1–25:1) [10–12]. In addition, Schanz and Juon regard the N:P ratio of 20:1 as a benchmark value for determining P-limited conditions in freshwater [13]. Guildford and Hecky proposed that P-only limitation occurs when the N:P ratio exceeds 22.6 in lake ecosystems [14]. A macroscale investigation of hundreds of lakes in the USA showed an average N:P ratio of ~54:1 in P-limited lakes [15]. In recent years, higher N:P ratios have been also observed in global large rivers, e.g., the Yangtze River (~53:1), the Han River (~65:1), and the Po River (~100:1) [16–18]. P is of fundamental importance for the synthesis of ATP, nucleic acids, phospholipids, and other key biomolecules [19]. The decreased availability of P may affect biogenesis of the cytoplasmic membrane, leading to ionic homeostasis disruption and changes in cell morphology [20]. Moreover, long-lasting P deficiency could cause severe repression of basic cellular processes, including carbon fixation, DNA replication, and protein biosynthesis [21], and even induce cell cycle arrest and apoptosis.

In natural systems, the spectrum of viral infection cycles is closely associated with nutrient supply and host availability [22]. Two diverging hypotheses within the framework of viral ecology reflect the complex scenarios in which environments select for more lytic or lysogenic infections. Analogous to the classic Lotka–Volterra model to explain the dynamics of predator and prey populations [23], “kill-the-winner” theory proposes a scenario in which lytic viruses prey preferentially on the most predominant hosts for reproduction of viral communities [24]. Conversely, “piggyback-the-winner” theory argues for the prevalent occurrence of lysogeny with the high-level bloom of host sources as a strategy for lysogenic viruses to adapt to environmental stimuli [25]. The controversy associated with viral lifestyles highlights the complicated virus–host interactions under different trophic statuses [22]. Previous studies on viral ecology assumed that lysogeny occurred more frequently in human guts and soils [26, 27], while lysis was more prevalent in cold deserts and oligotrophic ocean waters [28, 29], closely associated with microbial productivity and virus-to-host ratios [3]. Under P-limited conditions, viruses are inclined to undergo highly lytic cycles to suppress host proliferation or adopt a temperate manner to coexist with hosts. During the infection period, viruses show the potential to modify the host’s metabolic state through the activity of auxiliary metabolic genes (AMGs) [30]. In low-P regions, viruses have evolved to contain AMGs involved in P acquisition, which augment P utilization and P metabolic processes within host cells in favor of self-adaptation and survivability [31].

Pathogens can enter the human body through direct exposure, drinking water, or the food chain, causing severe diseases such as tuberculosis, cholera, and typhoid [32]. Conventional pathogen detection depends on microbial cultivation and biochemical identification [33], characterized by a long test cycle and complicated operation. Polymerase chain reaction (PCR)-based methods, as an alternative pathogen detection approach, may target only limited pathogenic species due to the specific design of PCR primers and validation processes [34]. With the increasing need for early warning and timely assessment of risks from pathogen transmission in water-related systems [35], metagenomic sequencing has been widely applied in water-quality surveillance for the rapid screening of pathogens at large scales [36, 37]. In particular, metagenome-assembled genomes (MAGs) provide fundamental resources for exploring the ecophysiology, microbial dynamics, and functions of uncultured microbes, including bacterial pathogens [38].

The Middle Route of the South-to-North Water Diversion Canal (MR-SNWDC) is the world’s largest artificial inter-basin water transfer engineering project for resolving water shortages in North China [39]. Since the operation of the MR-SNWDC in 2015, water-quality safety has become a major public concern, mostly in relation to water-quality constituents, nutrients, algae, microbial diversity, and other ecological drivers [40]. In the relatively closed MR-SNWDC, long-lasting P deficiency and P decline downstream strongly impacted viral/pathogen dynamics and consequently affected water-quality safety. Based on sequenced metagenomes sampled along the whole MR-SNWDC, we profiled the biogeographical patterns of viruses/hosts, as well as the spatial distribution and growth conditions of bacterial pathogens in spring and autumn. In addition, we examined multiple important environmental variables during our monitoring campaign, and revealed that P was the key environmental factor affecting viral and bacterial communities. We further explored the special ecophysiological traits of viruses and bacterial pathogens under P starvation. Unexpectedly, we discovered an interesting natural paradigm of water “self-purification” in the MR-SNWDC stretching a length of 1432 km, in which water-quality improvement benefitted from predation effects of indigenous viral populations resulting in recession of pathogenic bacteria from upstream to downstream along the canal. Using the newly recovered 4443 MAGs and 40,261 nonredundant viral operational taxonomic units (vOTUs), we highlighted the particular significance of virus–pathogen infection dynamics under extreme nutritional conditions in water self-purification and water resource protection.

## Materials and methods

### Data acquisition, sample collection, DNA extraction and sequencing

Historic records (2015~2021) on TP concentration were derived from China South-to-North Water Diversion Co., Ltd. (see Source Data), which were measured monthly at 32 monitoring stations distributed along the 1,432 km canal (Fig. S1). To obtain the matched information on abiotic and biotic constituents, special sampling campaigns were conducted at the same sites in August 2020 (autumn) and March 2021 (spring), with no impacts of extreme climates (Table S1). The length of the branching canal network between each pair of sampling sites, defined as the dendritic distance [41], was calculated using ESRI ArcGIS v10.2 software. Cumulative dendritic distance was calculated as the sum of all paths of the source water area to a sampling site, representing geographical attributes of the sampling sites. At each sampling site, over 30 L of water was collected with 4.5 L sterile polyethylene terephthalate (PET) bottles and transferred to the laboratory at 4 °C. Then, we filtered the 30 L water samples through 0.22 μm polycarbonate membranes (Millipore, USA) using a water-circulating multifunction vacuum pump (SHZ-D (III), Te’er Instrument and Equipment Co., Ltd, Zhengzhou, China) within 24 h.

Multiple important environmental factors were monitored at each sampling site, including pH, electrical conductivity (EC, μs/cm), turbidity (TUB, NTU), temperature (T, °C), fluoride ion (F^−^, mg/L), sulfate (SO_4_^2−^, mg/L), total organic carbon (TOC, mg/L), dissolved organic carbon (DOC, mg/L), permanganate (COD_Mn_, mg/L), ammonium nitrogen (NH_4_^+^-N, mg/L), nitrate nitrogen (NO_3_^−^-N, mg/L), total nitrogen (TN, total dissolved N plus total particulate N, mg/L), total phosphorus (TP, total dissolved P plus total particulate P, mg/L), and the N:P ratio (molar ratio of TN to TP, mol/mol). These regular environmental indicators are required in China’s Environmental Quality Standards for Surface Water (GB3838-2002) [42] or suggested by local water monitoring stations, representing the general nutrient conditions and water physiochemical quality. The standard GB3838-2002 classifies water quality into five categories (Classes I, II, III, IV, and V) from excellent (Class I) to poor (Class V), with proposed thresholds of minimum concentrations for key physicochemical constituents that would cause health risks. All the selected environmental factors were determined based on the standard methods recommended by the Ministry of Ecology and Environment of China [43]. The TP concentration in situ was measured using continuous flow analysis and the ammonium molybdate spectrophotometry method (HJ 670-2013) [44].

Extraction of total genomic DNA from samples was performed using the FastDNA Spin Kit for Soil (MP Biomedicals, USA) based on the manufacturer’s protocols. The concentration of extracted DNA products was determined with a TBS-380 fluorometer (TurnerBioSystems, USA). The relative fluorescence unit of each dilution was checked against the RL standard curve to calculate the DNA concentration in each sample. A NanoDrop2000 spectrophotometer (Thermo Fisher Scientific, USA) was employed to detect the purity of the extracted DNA products. The integrity of the DNA extracts was estimated using agarose gel electrophoresis. Specifically, DNA extract was added to a 1% agarose gel and then subjected to electrophoresis at 5 V/cm for 20 min to visualize the separated DNA fragments.

Sequencing libraries were generated by NEXTFLEX Rapid DNA-Seq (Bioo Scientific, Austin, TX, USA) following the manufacturer’s recommendations. The paired-end library was sequenced on the NovaSeq 6000 platform (Illumina Inc., San Diego, CA, USA) at Majorbio Bio-Pharm Technology Co., Ltd (Shanghai, China), using NovaSeq Reagent Kits, and 150 bp paired-end reads were finally obtained.

### Read processing and assembly

Sequenced paired-end reads were quality-controlled using TrimGalore v0.6.4 (--stringency 5; -e 0.1; --length 35; --paired; --max_n 0) within metaWRAP v1.2.2 [45]. MEGAHIT v1.1.3 [46] was used for de novo assembly with a minimum contig length of 1500 bp.

### Generation and taxonomic assignments of prokaryotic metagenome-assembled genomes

Contigs from each assembly were binned using the binning module within metaWRAP v1.2.2 [45] (--metabat2 --maxbin2 –concoct --run-checkm). The initially merged bins were checked by the bin-refinement module and identified as prokaryotic MAGs with completeness > 70% and contamination < 10%. The selected thresholds of MAGs were more rigorous than the medium-quality-level based on the Minimum Information about a Metagenome-Assembled Genome (MIMAG) criteria (completeness ≥ 50%, contamination < 10%) [47], and help provide both abundant and robust community-wide information for residential bacteria in the MR-SNWDC. To ensure reliability of the results, subsequent downstream analyses were also performed for high-quality genomes (completeness > 90%, contamination < 5%) in parallel. Taxonomic assignment of each MAG was conducted using GTDB-Tk [48] based on Genome Taxonomy Database (GTDB, http://gtdb.ecogenomic.org) Release 202.

### Identification and taxonomic classification of viral contigs

Viral contigs in each assembly were identified using viralVerify [49], VIBRANT v1.2.1 [50], DeepVirFinder v1.0 [51], PPR-Meta v1.0 [52], and Virsorter2 v2.1 [53]. Sequences with scores > 0.85 and *p* values < 0.05 were retained based on the DeepVirFinder tool. Then, CheckV v0.7.0 [54] was applied to estimate the completeness of all contigs identified with the five tools. Contigs containing provirus integration sites were first processed to remove host regions. The selection of putative viral contigs was based on the following criteria: (I) <90% completeness (low/medium-quality) and contig length ≥ 5 kb; (II) ≥90% completeness (high-quality and complete). Viral contigs identified from all assemblies were dereplicated and clustered at 95% average nucleotide identity (ANI) using CD-HIT v4.8.1 [55] (-c 0.95; -aS 0.85). The representative nonredundant sequences were denoted as vOTUs. Taxonomic annotations of vOTUs were performed using geNomad v1.3.0 (https://github.com/apcamargo/genomad). BACPHLIP [56] was applied to predict the lifestyles of vOTUs with high-quality or complete genomes.

### Abundance profiles

All MAGs were compiled into combined contigs. Bowtie2 v2.3.5.1 (http://bowtie-bio.sourceforge.net/bowtie2) was applied to build index of vOTUs/MAGs and perform read mapping against the vOTUs or contigs from MAGs. Sorted bam files generated by SAMtools v1.9 (http://samtools.sourceforge.net) were used to calculate coverage across samples using CoverM v0.6.1 (https://github.com/wwood/CoverM) (contig mode for vOTUs and genome mode for MAGs; -m rpkm; --min-read-percent-identity 95; --min-read-aligned-percent 90; --contig-end-exclusion 0; --no-zeros). The coverage value was expressed in the form of reads per kilobase per million mapped reads (RPKM) values [57].

### Host prediction

The virus‒host linkages were predicted via three complementary in silico methods, which were based on distinctive genomic characteristics of viruses and prokaryotes over their long-term interactions [58]. (I) Nucleotide sequence homology. During the long-term molecular coevolution process, viruses and hosts may develop many shared genomic signals for the exploration of virus‒host associations [59]. Sequence comparisons were performed between vOTUs and prokaryotic MAGs using BLASTn (≥90% minimum nucleotide identity, ≤0.001 e-value). (II) Transfer RNA (tRNA) match. Some viruses may show the adjustment of codon usage profiles to match tRNA genes to those of host genomes under evolutionary stress [60]. ARAGORN v1.265 [61] (‘-t’ option) was applied to identify tRNAs from prokaryotic MAGs and vOTUs, with subsequent alignments using BLASTn to meet the requirements of 100% sequence identity and 100% length coverage. (III) CRISPR spacer match. CRISPR represents an acquired prokaryotic immune mechanism to recognize and memorize short segments from the genome of the viral invader [62]. CRISPRs were identified from prokaryotic MAGs using minced v0.4.2 [63], and were then matched against viral contigs by BLASTn with ≤1 mismatch and 100% coverage, thus creating a virus‒host linkage. The results from the three methods were united to represent putative virus‒host associations. To ensure the reliability of host prediction, we used the reported virus‒host relationships in the Virus‒Host Database (virus‒host DB, available at https://www.genome.jp/virushostdb) as positive controls to remove taxonomically unmatched virus‒host linkages. The virus‒host pairs for the unclassified viruses were included for downstream analyses since there was insufficient evidence to exclude the possibility of associations between these viruses and their hosts [64]. The construction of the bipartite co-occurrence network of the virus‒host linkages is described in the Supplemental Methods.

### Resistome, virulence gene and pathogenic bacteria characterization

All open reading frames (ORFs) of MAGs were aligned to the SARG v2.2 database [65] and Virulence Factor DataBase (VFDB) [66] using BLASTp with an e-value ≤ 10^−5^. A protein sequence was annotated as an antibiotic resistance gene (ARG) or virulence factor gene (VFG) if the best hit showed an amino acid identity ≥ 80% and a query coverage ≥ 80% [67]. Prokaryotic MAGs with VFGs were regarded as potential pathogenic bacteria, and those carrying both VFGs and ≥10 copies of ARGs were considered “super pathogens” [36].

### Gene annotation of bacterial and viral genomes

Predicted proteins of MAGs and vOTUs were annotated by eggNOG-mapper [68] based on the bacteria dataset in the eggNOG database v5.0 [69], with the DIAMOND option and an e-value < 1e^−5^. Each annotated gene was assigned to a specific COG group. The prediction and validation of virus-encoded auxiliary metabolic genes (AMGs) are described in detail in the Supplemental Methods.

Single-copy genes were extracted using the fetchMG tool [70]. The number of each annotated gene was divided by the average numbers of 10 universal single-copy genes (COG0012, COG0016, COG0018, COG0172, COG0215, COG0495, COG0525, COG0533, COG0541, and COG0552) in each sample dataset, which represented the average gene copy number [71].

### Comparisons of viral genomic properties in the MR-SNWDC and the IMG/VR database

A total of 22,387 viral sequences with complete genomes were extracted from four representative surface water ecosystems, i.e., wastewater, marine, lake, and river, in the IMG/VR database [72]. The vOTUs in the MR-SNWDC with lengths over 5 kb and 100% completeness were selected to perform an unbiased comparison with publicly available viruses. The statistically significant differences in total scaffold size, CDS quantities, GC content, and amino acid composition of viral genomes in the MR-SNWDC and the IMG/VR database were evaluated using the Kruskal‒Wallis test and Bonferroni-corrected Wilcoxon test for multiple comparisons. Additional Supplemental Methods for estimation of the relationship between the vOTUs in the MR-SNWDC and publicly available viruses in the IMG/VR database are provided.

### Statistical analyses

All statistical analyses were performed using R 4.1.1 with a significance level of *p* < 0.05 unless otherwise mentioned.

Nonmetric multidimensional scaling (NMDS) was used to visualize the spatiotemporal distribution of viral and bacterial communities based on the Bray‒Curtis dissimilarity matrix generated from relative abundance tables for vOTUs or MAGs. Viral and bacterial communities were geographically clustered into four ecological regions from upstream to downstream along the canal, defined as Reg 1~4. The significance of spatiotemporal variation was estimated by permutational multivariate analysis of variance (PERMANOVA).

The matrices of pairwise dendritic distances were transformed into principal components of neighborhood matrices (PCNMs) using the pcnm function in the “vegan” R package [73]. The resulting PCNMs 1~3 with positive eigenvalues were selected for the subsequent analysis. A partial Mantel test (PCNM-corrected) was applied to estimate the correlations between multiple environmental factors and Bray‒Curtis distances calculated from the relative abundances of vOTUs or MAGs. We further performed random forest analysis to examine the major environmental drivers of viral and bacterial communities. The importance of each environmental factor was represented by the increase in node purity or the percentage increase in the mean squared error (MSE), with higher values implying more important factors [74]. The significance level of each environmental factor was assessed based on 5000 permutations using the “rfPermute” R package [75]. In addition, we performed co-occurrence network analysis to examine the influence of environmental factors on MAGs/vOTUs, which facilitated interpretation of the system-level properties of microbial communities from the perspectives of robustness and modularity [76]. The networks were constructed based on Spearman’s correlations between environmental gradients and MAG/vOTU abundance. MAGs or vOTUs with relative abundances in all samples less than 0.001 were excluded from the analyses. Only the linkages with correlation coefficients ≥ 0.7 and Bonferroni-adjusted *p* values < 0.05 were visualized in the networks [77].

To estimate the proliferation dynamics of bacterial populations from the source water area to downstream regions, a regional growth factor (RGF) was defined, analogous to the previously defined local growth factor (LGF) for microbial communities [40]. The absolute abundances were averaged within each region, with subsequent comparison of abundances in Reg 2~4 and the water source area (Reg 1), which was as follows:

1 $${{{{{\rm{RGF}}}}}}_{x,i} = {{{{{\rm{log10}}}}}}\frac{{{{{{{\rm{ABUN}}}}}}_{{{{{{\rm{Reg}}}}}}_{x,i}} + 1}}{{{{{{{\rm{ABUN}}}}}}_{{{{{{\rm{Reg}}}}}}_{1,i}} + 1}}$$

where ${{{{{\rm{ABUN}}}}}}_{{{{{{\rm{Reg}}}}}}_{x,i}}$ is the absolute abundance of MAG_*i*_ in Reg_x_ and ${{{{{\rm{ABUN}}}}}}_{{{{{{\rm{Reg}}}}}}_{1,i}}$ is the absolute abundance of MAG_*i*_ in Reg 1. Within each region, bacterial populations were classified into four categories, including “emerged” (RGF > 0 and ${{{{{\rm{ABUN}}}}}}_{{{{{{\rm{Reg}}}}}}_{1,i}} = 0$), “promoted” (RGF > 0 and ${{{{{\rm{ABUN}}}}}}_{{{{{{\rm{Reg}}}}}}_{1,i}} > 0$), “inhibited” (RGF < 0 and ${{{{{\rm{ABUN}}}}}}_{{{{{{\rm{Reg}}}}}}_{x,i}} > 0$), and “vanished” (${{{{{\rm{ABUN}}}}}}_{{{{{{\rm{Reg}}}}}}_{x,i}} = 0$).

## Results

### Spatiotemporal distribution of viral and bacterial communities

**Fig. 1:**
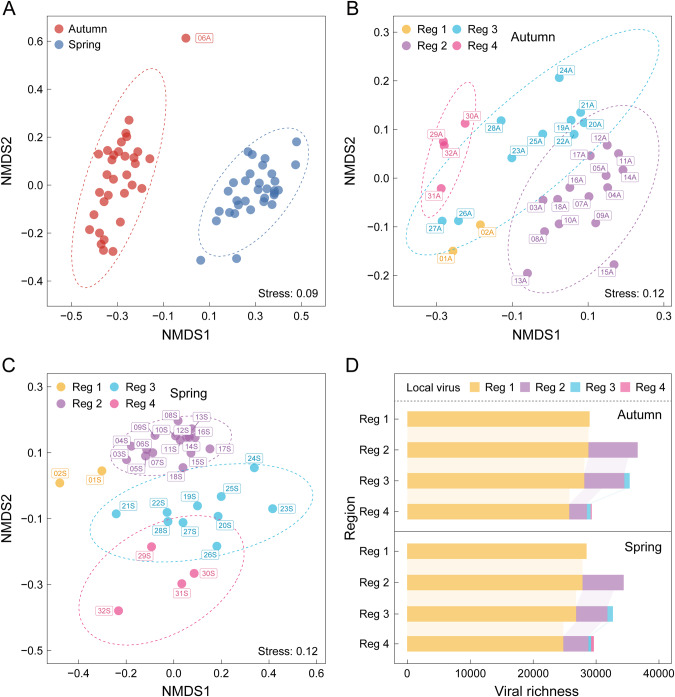
Spatiotemporal distribution of viral communities in autumn and spring. Nonmetric multidimensional scaling (NMDS) analyses visualize the temporal variation of viral β-diversity (**A**) as well as the distinct partition into four ecological regions of viral communities in autumn (**B**) and spring (**C**), based on the Bray–Curtis dissimilarity matrix calculated from the relative abundances of vOTUs. The stress value denotes the ordination fitness of each NMDS plot. Each group is encircled by an ellipse at 95% confidence interval. One outlier sample (06A) is excluded from subsequent analyses. **D** Sankey plots display the distribution of vOTUs from the water source area (Reg 1) to the water-receiving areas (Reg 4). The bars in different colors represent the number of vOTUs from the local region (firstly appeared in the concerned region but were not detected in the upper reach of the region). Source data are provided in the Source Data file.

A total of 5.61 Tb of metagenomic data were derived from 64 samples taken at 32 monitoring sites along the MR-SNWDC in spring and autumn (Fig. S1 and Table S1). The 40,261 nonredundant vOTUs (with an average length of 16.4 kb) from the viromic analyses were assigned to four genome quality levels, including complete (1.6%), high-quality (3.2%), medium-quality (4.7%), and low quality (90.5%) (Table S2). A large fraction of low-quality viral genomes appeared in the IMG/VR database [72], the Global Ocean Virome (GOV) 2.0 dataset [78], and other relevant datasets from studies on viral ecology [4, 57], reflecting the novelty of environmental viruses with low similarity to currently limited viral reference genomes as well as the challenge of assembling complete viral genomes using short-read metagenomes. Among the 30,453 vOTUs assigned putative taxonomy, 99.7% were affiliated with double-stranded DNA (dsDNA) viruses and 92 vOTUs were single-stranded DNA (ssDNA) viruses. Although NMDS analysis showed significant clustering of viral β-diversity according to autumn (2020) and spring (2021) (*p* < 0.001, Fig. 1A and Table S3), more time-series data (*N* > 2) might be needed to support the seasonal shifts in viral communities. Four distinct ecological regions emerged for the endemic spatial distribution of viral communities (*p* < 0.001, Fig. 1B, C, and Table S3), defined as Reg 1 (Danjiangkou Reservoir: 01–02), Reg 2 (upstream: 03–18), Reg 3 (downstream: 19–28), and Reg 4 (water-receiving areas: 29–32).

Since the cement boundary of the channel largely blocks the connectivity to external surface or subsurface runoff, the MR-SNWDC is regarded as an ideal closed unidirectional flow system with downstream microbial species mostly derived from the upstream reservoir. The β-diversity of viral communities showed high Sorenson similarity (>0.85) between the four regions, indicating high concordance in the frequency of viral occurrence in the whole canal (Fig. S2). Furthermore, the spatial distribution of viral populations from upstream to downstream demonstrated that approximately 75% of vOTUs in the whole MR-SNWDC were observed first in the Danjiangkou Reservoir, specifically accounting for 75~90% of viral richness in each of the three concerned main-canal regions (Fig. 1D). The spatiotemporal pattern of viral communities was consistent with that of bacterial communities represented by 4443 MAGs, although a general balance of viral richness was maintained with a significant loss of bacterial richness downstream (Fig. S3).

### P limitation as a major factor influencing viral and bacterial communities

**Fig. 2:**
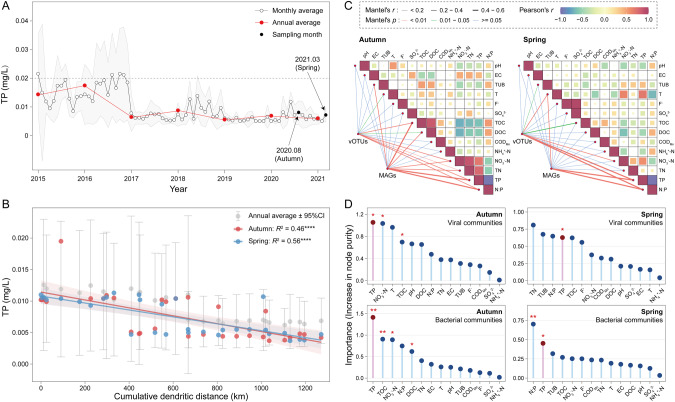
Longitudinal dynamics of TP concentration in the MR-SNWDC as well as relevance of environmental factors and viral/bacterial communities. **A** Monthly and annual average TP concentrations from 2015 to 2021. The gray area displays standard deviation of monthly average TP content. **B** Longitudinal changes in TP concentration along the canal. The gray dots represent the annual average TP concentration across 7 years. The error bar corresponds to the 95% confidence interval. The red and blue dots represent the TP concentration in the sampling months. The goodness-of-fit *R*^2^ value and the statistical significance are presented for each linear regression (****: <0.0001). **C** Correlation between environmental factors and viral/bacterial communities in autumn and spring. Pairwise Pearson’s coefficients are denoted by color gradients. Edge width demonstrates the Mantel’s *r* correlation coefficients. Edge color represents the significance level of *p* value based on 999 permutations. **D** Random forest importance of each environmental factor for viral and bacterial communities in two seasons. All environmental factors are brought into a ranking by their importance index represented by the increase in node purity. The significance of each environmental factor is shown in asterisks (**: <0.01; *: <0.05). Source data are provided in the Source Data file.

Over seven years (2015~2021) monthly water quality monitoring provided evidence of long-lasting P limitation in the MR-SWNDC, with almost all the spatially or temporally measured TP concentrations lower than the P-oligotrophic criteria of 0.02 mg/L. According to China’s Environmental Quality Standards for Surface Water (GB3838-2002) [42], the observed TP < 0.02 mg/L meant that water quality was ranked as the best (Class I) for drinking water purposes. The annually averaged TP concentration in the last three years (2019~2021) ranged between 0.006 and 0.007 mg/L in the MR-SNWDC (Fig. 2A), lower than that in other representative river or lake ecosystems from the Global Freshwater Quality Database (United Nations Environment Programme, GEMStat, https://gemstat.org/), which covered TP records in 2015~2021 and at least one year of monitoring data in 2019~2021 (Table S4).

Biologically important elements such as C, N, and P play fundamental roles in the physiological activities of microorganisms. For example, both heterotrophs and phototrophs require N and P for cellular components and enzymatic activities, despite of their differentiation in carbon utilization and lifestyles. In the present study, the measured TOC and TN concentrations were maintained at relatively stable levels along the canal. However, the coefficient of variation (the standard deviation divided by the mean) of TP was 0.48 and 0.39 in autumn and spring, respectively, approximately 2~5-fold higher than that of TOC (autumn: 0.12, spring: 0.11) or TN (autumn: 0.10, spring: 0.06), suggesting that the MR-SNWDC was more P-sensitive than C- or N- sensitive. The extremely low-P content (0.008 mg/L in autumn and 0.007 mg/L in spring) (Fig. 2A) resulted in a very high average N:P ratio (~350:1) relative to the reference N:P level (16:1) of the Redfield ratio (Fig. S4), indicating P-starvation conditions in the MR-SNWDC. Based on the 7-year monitoring data, the high N:P ratio was sustained over the long term (290:1~405:1) along the main canal (Fig. S5).

Moreover, the spatial variation in TP concentration obtained from the special monitoring campaigns (August 2020 and March 2021), similar to the trend of annually averaged TP content along the main canal across seven years, showed a significant decrease from 0.010~0.011 mg/L in the upper reach within the source water area to 0.004 ~ 0.005 mg/L in the lower reach near the Beijing and Tianjin municipalities (autumn: *R*^2^ = 0.46; spring: *R*^2^ = 0.56) (Fig. 2B). Since the closed system with cement boundary of the channel significantly reduced the external input of P pollution, the main P supply in the whole canal was likely to be derived from phosphate fertilizer in the basins around the water source area (Danjiangkou Reservoir) [79], suggesting similar decline trends in the TP concentration level and P flux downstream (Fig. S6) due to the continuous dilution and microbial consumption.

We further employed environmental correlation analysis, a random forest model, and co-occurrence network analysis to uncover the major effects of TP on viral and bacterial communities based on 14 environmental indicators recommended by China’s Environmental Quality Standards for Surface Water (GB3838-2002) and local water monitoring stations. Considering that carbon source might be a more direct growth-limiting factor for heterotrophs [80], we also performed the same analyses for heterotrophic bacteria by excluding phototrophs (mostly *Cyanobacteria*) from the community datasets. The partial Mantel test demonstrated that TP and the N:P ratio were highly correlated with vOTUs or MAGs in both autumn and spring (Figs. 2C and S7A). Using the random forest model, we analyzed the importance of each environmental factor and found that TP was the most significant environmental factor influencing the viral and bacterial communities in both seasons (Figs. 2D and S7B). Bacterial communities in spring were highly influenced by the N:P ratio, which was largely a P-limited process since N was generally sufficient and non-sensitive in the MR-SNWDC. In addition, the co-occurrence network of environmental factors and MAGs/vOTUs showed that TP and N:P were present in the largest module with the most connections to MAGs/vOTUs in both seasons, suggesting their dominant effects on bacterial and viral communities (Fig. S8).

### Viral genomic adaptation to P-limited environments

**Fig. 3:**
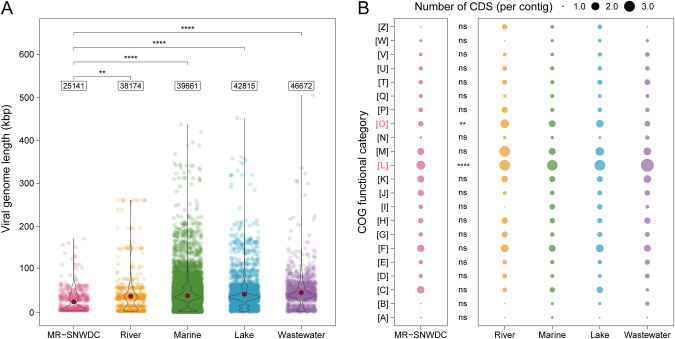
Genome size and CDS quantities of viruses with complete genomes in the MR-SNWDC and the IMG/VR database. **A** Genome length of viruses in the MR-SNWDC and four representative surface water ecosystems (river, marine, lake, and wastewater) from the IMG/VR database. The average viral genome length of each dataset is marked by the red dot and denoted in the label. The statistical difference is estimated by Bonferroni-adjusted Wilcoxon test. **B** Average quantities of viral CDSs in each COG functional category. Kruskal–Wallis test is performed for differences of viral CDSs in the MR-SNWDC and the IMG/VR database (combining four ecosystems). All above significance levels are marked by asterisks (****: <0.0001; **: <0.01; ns: >0.05). The COG functions include: A (RNA processing and modification), B (Chromatin structure and dynamics), C (Energy production and conversion), D (Cell cycle control, cell division, chromosome partitioning), E (Amino acid transport and metabolism), F (Nucleotide transport and metabolism), G (Carbohydrate transport and metabolism), H (Coenzyme transport and metabolism), I (Lipid transport and metabolism), J (Translation, ribosomal structure and biogenesis), K (Transcription), L (Replication, recombination and repair), M (Cell wall/membrane/envelope biogenesis), N (Cell motility), O (Posttranslational modification, protein turnover, chaperones), P (Inorganic ion transport and metabolism), Q (Secondary metabolites biosynthesis, transport and catabolism), T (Signal transduction mechanisms), U (Intracellular trafficking, secretion, and vesicular transport), V (Defense mechanisms), W (Extracellular structures), and Z (Cytoskeleton). Source data are provided in the Source Data file.

Compared with the average size of the 22,386 complete viral genomes under a broader P range in four representative surface water ecosystems of the IMG/VR database, the genome size (average value of 25 kb) of the 646 vOTUs with complete genomes (see Table S2) derived from the extreme P-constrained environment in the MR-SNWDC was apparently smaller than that in lakes, marine environments, or rivers, and much smaller than that in P-rich wastewater (average value of 47 kb) (Fig. 3A). The TP concentration for these selected public biomes (if reported) was over 0.02 mg/L, higher than the upper bound of TP content in the MR-SNWDC. The small genome size was mainly attributed to the reduced encoding frequency of functional genes associated with genome replication, recombination, and repair, as well as posttranslational modification, protein turnover, and chaperones (Fig. 3B). Moreover, the viruses under P-constrained conditions contained higher guanine-cytosine (GC) contents (Fig. S9A) and more specific amino acid residues related to protein stability (Fig. S9B) than those in the IMG/VR database, suggesting a general robustness of viral structures in the MR-SNWDC. The lower lysine-arginine ratio and greater proline content of viral genomes in the MR-SNWDC helped interpret the improved emergence of salt bridges and hydrogen bonds between the secondary protein structures [81], which served as the main driver to enhance viral stability. Asparagine, methionine, and tyrosine, which are closely associated with protein-folding flexibility and are usually involved in optimization under cold conditions [4], showed significantly lower frequencies in viral genomes in the MR-SWNDC, suggesting a reduction in flexibility factors in the viral molecules. More information on the relationships between vOTUs in the MR-SNWDC and publicly reported viral sequences in the IMG/VR database is provided in the Supplemental Results and Fig. S10.

### Repressed growth potential of bacterial communities under P limitation

**Fig. 4:**
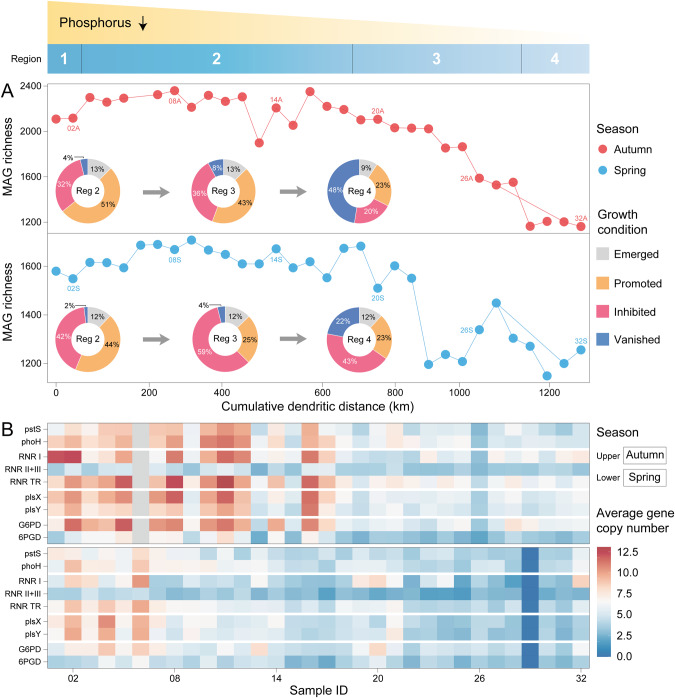
Richness, growth potential, and P-associated functional capabilities of bacterial communities in the MR-SNWDC. Thirty-two sampling sites are arranged along the 1432 km canal which is classified into four distinct regions (Top graph). The cumulative dendritic distance is calculated between each sampling site and the water source area (see “Materials and methods”). **A** Line charts demonstrate changes in the richness of bacterial populations represented by MAGs along the canal, while pie charts indicate proportional variation in the emerged, promoted, inhibited, and vanished bacteria in three main-canal regions (see “Materials and methods”). **B** Heatmap showing average copy number of key bacteria-encoded P-associated genes across sampling sites. The upper and lower panel represent the autumn and spring season, respectively. pstS phosphate transport system substrate-binding gene, phoH phosphate starvation-inducible gene, RNR ribonucleotide reductase (I: *nrdABEFHI*, II: *nrdJ*, III: *nrdDG*, TR transcriptional regulator *nrdR*), plsX phosphate acyltransferase, plsY glycerol-3-phosphate acyltransferase, G6PD glucose-6-phosphate 1-dehydrogenase, 6PGD 6-phosphogluconate dehydrogenase. Source data are provided in the Source Data file.

A general decreasing trend in bacterial richness was observed along the canal with the decline in TP concentration downstream in both spring and autumn (Fig. 4A), notably in autumn. The residential bacterial MAGs (2109) originating from the water source area (Danjiangkou Reservoir) were reduced by approximately half at the canal end (water-receiving areas) in autumn. Despite the overall decline in the number of MAGs with water flow, we noticed a slight increase in bacterial richness from Reg 1 to Reg 2, reflecting the potential introduced external species from surface/subsurface runoff in the wet season along Reg 2 stretching ~660 km. Comparison of the bacterial abundances in the main canal and water source area revealed proportional variation in the emerged, promoted, inhibited, and vanished bacterial populations along the MR-SNWDC (Fig. 4A, see “Materials and methods”). In both seasons, approximately 60% of bacterial MAGs showed emergence or bloom cycles in Reg 2, followed by a subsequent drop in Reg 3 and Reg 4. The proportion of vanished bacteria was 4~10 times greater in the water-receiving areas than in Reg 2 or Reg 3, corresponding to the significant decrease in P supply and strong environmental filtering effects on the die-off patterns of populations with low adaptation.

Some core phosphate regulon genes, especially the phosphate transport system substrate-binding *pstS* gene and phosphate starvation-inducible *phoH* gene, play significant roles in P acquisition activities [82]. These genes showed a noticeable decline (*R*^2^ = 0.39, *p* < 0.0001) in encoding frequencies along the canal (Fig. 4B and Fig. S11). Meanwhile, a significant reduction in average copy number of other key P-based genes was also observed in the flow direction, which was essential to nucleotide synthesis, DNA replication, and membrane formation processes, including the pentose phosphate pathway (PPP: *G6PD*, *6PGD*) (*R*^2^ = 0.32, *p* < 0.0001), phospholipid biosynthesis (*plsX*, *plsY*) (*R*^2^ = 0.46, *p* < 0.0001), and ribonucleotide reductase (RNR: RNR I + II + III, RNR transcriptional regulator) (*R*^2^ = 0.30, *p* < 0.0001). Similar trends in richness, growth potential, and gene variation were observed for the high-quality-level bacterial genomes (completeness > 90%, contamination < 5%), confirming the reliability of the results (Fig. S12). In addition to the P-based genes, we investigated other functional genes associated with carbohydrate, energy, nitrogen, sulfur, and nucleotide metabolism, and found a consistent decline in the average copy number of these genes along the canal (Fig. S13). The extremely low-P supply in downstream regions may fall outside the range of P contents in which bacteria can maintain normal physiological activities, thus showing weakened biological functions related to P acquisition and other basic cellular processes. The universally inhibited presence of P-associated genes, as well as the repressed richness and growth potential of bacteria, suggested their low environmental fitness under selection driven by P constraints.

### Special virus‒host dynamics under P constraints

**Fig. 5:**
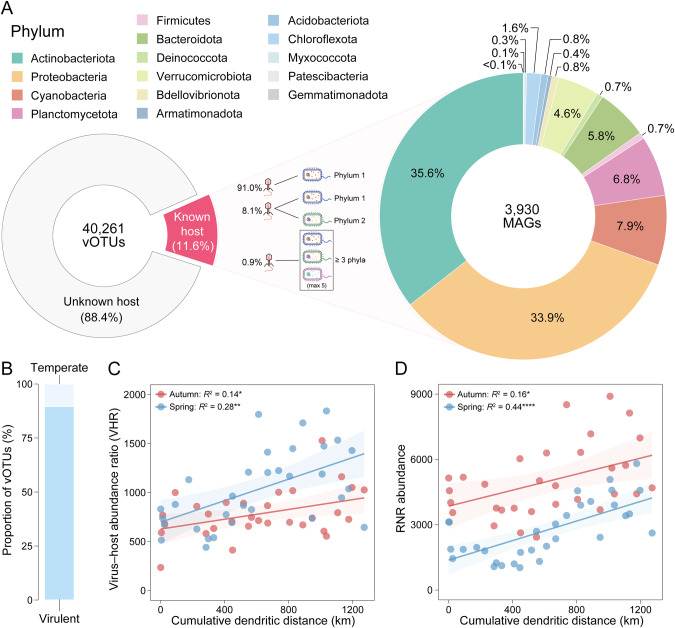
Virus–host linkages and viral infection patterns. **A** Virus–host linkages display viral host range profiles spanning diverse bacterial phyla, with most vOTUs linked to single host phylum and minor vOTUs exhibiting cross-phyla (≥2) relationships. **B** Lifestyle prediction provides the proportion of vOTUs (with complete genome) adopting virulent or temperate infection cycles. Both seasons witness significant increasing trends in **C** virus–host abundance ratio (VHR) and **D** virus-encoded RNR-associated gene abundance along the canal. The goodness-of-fit *R*^2^ value and the statistical significance are presented for each linear regression (*: <0.05; ***: <0.001; ****: <0.0001). Source data are provided in the Source Data file.

Based on in silico prediction and positive controls using known virus‒host associations, 11.6% of the total 40,261 vOTUs were assigned to putative prokaryotic hosts spanning 15 bacterial phyla, in which the majority (91.0%) were linked to only a single host phylum but some (0.8%) exhibited a broad host range across 3 or more phyla (max 5) (Fig. 5A and Table S5). These host-assigned vOTUs accounted for approximately 60~90% abundances of the total vOTUs in all samples. Among 33,391 paired virus‒host linkages, *Actinobacteriota* accounted for the largest portion in the number of hosts (35.6%), followed by *Proteobacteria* (33.9%) (Fig. 5A). Among taxonomically known viruses within validated virus‒host linkages, approximately 99.9% belonged to *Caudoviricetes*, which represented the most abundant dsDNA phages, as well as 17 ssDNA phages (*Inoviridae*). For each bacterial phylum, viral abundance showed a significant positive correlation with host abundance, suggesting the accuracy of the identified virus‒host linkages (Fig. S14). Virus‒host abundance ratios (VHRs) were greater than one (16.7~2021.4) for all bacterial phyla (Fig. S15). A total of 4659 vOTUs and 3930 MAGs were visualized in the co-occurrence network (Fig. S16). All virus‒host linkages were clustered into 153 distinct separated modules. The five largest representative modules showed the most compound linkages between viruses and their hosts, with over 400 community members in each module. Host co-occurrence and broad host range of viruses helped interpret the diverse and complex virus‒host cross-infections in these modules. Approximately 23.5% of modules represented linkages between a single virus and a host strain only.

Among the 1945 viruses with high-quality or complete genomes, ~90% were predicted to adopt a lytic lifestyle (Fig. 5B). Increased VHRs towards downstream (autumn: *R*^2^ = 0.14, *p* < 0.05; spring: *R*^2^ = 0.28, *p* < 0.01) reflected an elevated level of viral infection cycles along the canal (Fig. 5C). Virus-encoded RNR genes displayed a significant increase in average abundance along the canal (autumn: *R*^2^ = 0.16, *p* < 0.05; spring: *R*^2^ = 0.44, *p* < 0.0001), consistent with the enhancement of infection dynamics (Fig. 5D), and may be linked to the promoted genome replication process for rapid reproductive assurance.

In this study, a large portion of virus-encoded AMGs of the *pstS* gene (refer to Supplemental Methods) were unveiled to indicate the capability of viruses to promote P acquisition of host cells under P-limited conditions (highlighted in bold font in Table S6). In addition to the expected presence of *pstS* [31], DRAM-v annotations provided information on other highly enriched AMGs associated with nucleotide metabolism, including dUTP pyrophosphatase (*DUT*), DNA (cytosine-5-)-methyltransferase (*DNMT*), dCTP deaminase (*dcd*), and thymidylate synthase (*thyA*), suggesting viral capacity to facilitate host metabolism in relation to nucleotide biosynthesis and DNA repair (Fig. S17A). In addition, we identified 54 AMGs as UDP-glucose 4-epimerase (*galE*) capable of providing the carbon flux and cofactors necessary for nucleotide-based catalytic activity. All selected representative AMGs were validated by modeling tertiary protein structures (Fig. S17B).

### Effects of viral predation on pathogen recession under extreme P limitation

**Fig. 6:**
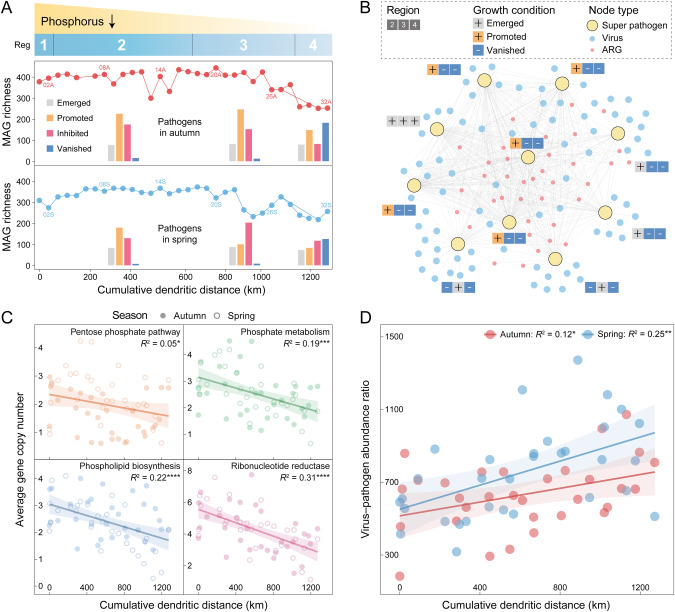
Dynamics and P-associated functions of bacterial pathogens, as well as their relationships with viruses. **A** Along the MR-SNWDC (1432 km) with decreasing P supply, bacterial pathogen populations, represented by MAGs, exhibit consistent trends in the richness (line charts) and growth potential (bar charts, see Materials and Methods) in autumn and spring. **B** The bipartite network represents the linkages between ten ARG-carrying super pathogens (see Materials and Methods) and their viral predators. Filled squares denote the emergence/proliferation (+) or vanishment (−) of each specific super pathogen in three main-canal regions. **C** Key pathogen-encoded genes of four metabolic processes, associated with P acquisition and utilization, show a significant decline in average copy number along the canal. **D** Virus–pathogen abundance ratios display a notable increase with water flow in both seasons. Each linear regression is denoted by the goodness-of-fit *R*^2^ value and the significance level of *p* value. Source data are provided in the Source Data file.

Pathogen-induced risk, as one of the public health issues of drinking water, is a major concern for water safety in the MR-SNWDC. Here, we investigated the spatiotemporal distribution and growth potential of pathogenic bacteria along the canal. In the water source area (Reg 1), 387 and 292 bacterial pathogens were initially detected in autumn and spring, respectively, accounting for 15.3% of all MAGs, with a general decline along the canal from the Danjiangkou Reservoir to the water-receiving areas (Fig. 6A). Although the whole canal is an artificially controlled system, external pathogenic species along Reg 2 stretching ~660 km were still potentially introduced with surface/subsurface runoff in the rainy season and resulted in a slight local increase in pathogen richness. The proportion of newly emerged or proliferated pathogens declined from Reg 2 (>60%) to Reg 4 (<40%), and the number of vanished pathogens in Reg 4 was almost tenfold of that in Reg 2 (Fig. 6A). Meanwhile, ten bacterial pathogens carrying multiple ARGs, as antibiotic-resistant super pathogens, appeared upstream but were almost eliminated in the water-receiving areas (Fig. 6B), suggesting a reduction in the current health risks posed by antibiotic-resistant pathogens.

With the decline in TP concentration downstream, the repression of pathogens’ functional potential associated with P metabolism (*R*^2^ = 0.19, *p* < 0.001), PPP (*R*^2^ = 0.05, *p* < 0.05), phospholipid biosynthesis (*R*^2^ = 0.22, *p* < 0.0001), and RNR (*R*^2^ = 0.31, *p* < 0.0001) was observed along the canal (Fig. 6C), which failed to ensure the normal bioactivities of P acquisition, nucleotide replication, and membrane formation, and further caused P-constrained cellular damage. In addition to P filtration, enhanced viral infections could also contribute to the diminishing of bacterial pathogens, as indicated by the significantly increased virus–pathogen abundance ratios along the canal (autumn: *R*^2^ = 0.12, *p* < 0.05; spring: *R*^2^ = 0.25, *p* < 0.01, Fig. 6D). These results stressed viral predation effects on pathogen recession under P limitation and provided evidence of reduced health risks from the water source area to the water-receiving areas in the MR-SNWDC.

## Discussion

As a rare case of natural water self-purification discovered in a large-scale aquatic system, the virus–pathogen dynamics demonstrated the great potential of viral predations by reducing over 30% of waterborne pathogens in the MR-SWNDC, accompanied by nearly complete elimination of the widely concerning antibiotic-resistant super pathogens in the water-receiving areas (Fig. 6).

**Fig. 7:**
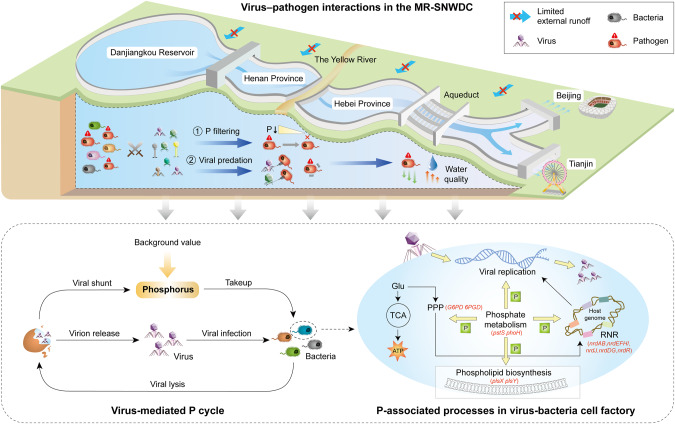
Schematic diagram of pathogen dynamics and P cycle along the 1432 km continuum in the MR-SNWDC. The MR-SNWDC originated from the Danjiangkou Reservoir, passing through Henan and Hebei Province, diverts water into the Beijing and Tianjin municipalities. Bacteria including pathogens act as one of the major consumers of P nutrients in the canal. Some core P metabolism genes (e.g., *pstS*, *phoH*) facilitate the P acquisition and provide P flux for other P-based processes associated with nucleotide sugar biosynthesis (PPP), membrane formation (phospholipid biosynthesis), and DNA replication (RNR). In the MR-SNWDC with long-lasting P deficiency and rapid decay of P supply along the canal, pathogens show the repression of environmental fitness, whereas viruses enhance their lytic infections for survivability. Viral predation causes bacteria mortality to release next-generation virions, and induces viral shunt of intracellular P for the turnover of other heterotrophic bacteria, which maintains sustainable host sources ready for next round of infection. The unique virus–pathogen infection dynamics under P constraints cause pathogen recession and improve water quality in the water-receiving areas.

Unlike other aquatic ecosystems, the MR-SWNDC encountered long-lasting P constraints (TP ≤ 0.02 mg/L and N:P 290:1~405:1) along the canal according to the monthly monitoring data from 2015 to 2021 (Figs. 2A, S5, and Source Data). As a bio-essential element of genomes [83], P scarcity affects pathogens’ metabolic processes and theoretical upper bound on growth potential. Normal cellular proliferation depends on sufficient storage of genetic materials, i.e., nucleotides, with the architecture of P elements (Fig. 7), while RNR functions as a key enzyme of the only known de novo synthesis pathway for deoxyribonucleoside triphosphate (dNTP) [84], the essential DNA precursor for nucleotide replication. The amount of RNR protein was found to be directly proportional to the DNA synthesis rate and played a crucial role in regulating cellular growth conditions during the generation time of *Escherichia coli* [85]. The extremely low P may have enhanced the decrease in RNR-associated genes along the canal (Fig. 6C), and induced imbalances in dNTP pools and increases in genome mutation [86]. In fact, RNRs have been applied in the target design of inhibitors to eliminate pathogenic infections [87]. Moreover, genome dysfunction was also reflected by the decreasing activity of the PPP (Fig. 6C), which reduced the necessary supply of carbon (ribose 5-phosphate) for nucleotide metabolism [88]. Additional hazards caused by P constraints could be attributed to the loss of membrane components contributing to cell malformation and a distorted intracellular environment [20] associated with the repression of rate-limiting genes (*plsX*, *plsY*) [89] involved in phospholipid biosynthesis. Thus, the decay of P availability in the MR-SNWDC highly affected the viability of pathogens (Fig. 7), and could significantly alleviate the pathogen-induced health risks from the water source to the water-receiving areas. The rigid boundary of the water transfer system largely prevented exogenous pollutants and pathogenic species from entering the channel except in the flood season, when surface runoff may introduce slight external inputs, especially in the longest span of Reg 2 in the MR-SNWDC.

In contrast, viruses, as another competitor for limited P resources, exhibited relatively strong adaptability and survivability through a “self-conservation” strategy. More specifically, viruses in the MR-SNWDC wisely adopted small genome size, almost half the average length of viral genomes from P-rich wastewater (Fig. 3A), to promote the efficiency of P utilization as well as to minimize the potential costs of nucleotide replication and DNA repair (Fig. 3B). Only the most necessary genetic materials were likely to be maintained to meet the minimal requirements for posttranslational modification processes such as protein phosphorylation [90]. To cope with the potential P-constraint stimulus, viruses need to enhance their molecular stability by increasing the presence of hydrogen bonds and salt bridges, indicated by increasing GC contents as well as encoding preferences for specific amino acids (Fig. S9). Viruses in a P-oligotrophic environment might tend to conserve limited energy, while cold-stressed viruses [4] would be required to move for energy production and thus likely to undergo adaptation for increased structural flexibility and protein folding to promote enzymatic activities under thermodynamically unfavorable conditions.

Viruses in the MR-SNWDC exhibited a typical way of obtaining P nutrients and energy from host cells through enhanced infection (Figs. 6D and 7). The increasing abundance of virus-encoded RNRs along the canal (Fig. 5D) facilitated the supply of dNTP substrate for DNA polymerization of viruses, suggesting enhancement of the competitive capability of viruses under P-limited conditions [91]. The regulation of RNR genes helped maintain a balance between the provision of DNA precursors and the requirements of viral production. The prevalently occurring virulent viruses (Fig. 5B) destroyed host cells for the release of next-generation virions (Fig. 7), exerting top-down control by suppressing the productivity of host populations [23]. Efficient viral predation for targeted elimination of bacterial pathogens helped improve water-quality safety in the canal (Fig. 7). Previous studies assessed viral contributions to biogeochemical pools and indicated that high lysis may promote viral shunt effects by releasing nutrient compounds within host cells for P reutilization [92, 93], which might be the only source for microbial turnover under extreme P constraints [94]. Quantitative estimates of the relative contributions of viral shunts to the P pool would require more sufficient P data and more rigorous biophysical models in the future. Virus-mediated P recycling provided positive feedback for maintaining a balance in the richness of viral communities, consistent with the experimental results [95] with respect to the promotion of viral production in a low-P environment. Moreover, viruses also encoded a large repertoire of AMGs associated with nucleotide metabolism (Table S6), showing high potential to affect host genome biosynthesis to promote self-proliferation [96].

The emergence and transmission of environmental pathogens have contributed to the global prevalence of human diseases [97]. Viruses, as natural predators of bacterial pathogens, can infect their hosts and cause host cell death through viral lytic cycles, exhibiting great potential in the treatment of pathogen-induced diseases. So-called “bacteriophage therapy” [98], capable of highly accurate and targeted treatments through specific viral infections and constant virus‒host coevolution, has been successful in therapeutics and aquaculture [99, 100]. Unlike strain-level targeted bacteriophage therapy in biomedical practices (Fig. S18), large repertoires of aquatic viruses with diverse infection spectra could simultaneously achieve effective elimination of multiple bacterial pathogens, which is particularly helpful for addressing the new challenges in drinking water safety related to pathogen outbreaks or dissemination of antibiotic-resistant microorganisms [36].

## Conclusions

We discovered an interesting natural paradigm of bacteriophage therapy for water-quality improvement via virus–pathogen infection dynamics in the world’s largest water transfer engineering project. We further interpreted the conditions necessary for the emergence of such a rare event in the MR-SNWDC, stretching 1432 km continuum, in which P limitation was sustained during a 7-year period (TP < 0.02 mg/L and N:P 290:1~405:1) and played a critical role in in situ viral and bacterial communities. We found that residential viruses have evolved smaller genomes to minimize nucleotide replication, DNA repair, and posttranslational modification costs under extreme P constraints. Moreover, we revealed the main structural driving forces enhancing viral stability under P-depleted conditions, mostly facilitated by an increased GC content as well as an encoding preference for amino acid residues associated with protein rigidity. With a decreasing P supply along the canal, bacterial pathogens exhibited weakened capability of critical P-based genes involved in P metabolism, membrane formation, and nucleotide biosynthesis, showing a loss of environmental fitness and significant recession downstream. Our study also unveiled the survival strategy of virulent viruses to secure P nutrients and energy from host cells by enhancing lytic infections and increasing the abundance of RNR genes linked to the promotion of nuclear DNA replication cycles, which reduced health risks from bacterial pathogens in the MR-SNWDC. This study provided an alternative pathway of water-quality improvement through natural viral predation, and highlighted the potential significance of self-purification associated with virus‒host interactions in drinking water protection and sustainable water resource management.

## Supplementary information

Supplementary Information

Supplementary tables

source data

## Data Availability

Raw sequencing metagenomes of the MR-SNWDC have been deposited in NCBI BioProject database under accession code PRJNA881510. Publicly reported viral sequences from other ecosystems used in this study are available in the IMG/VR database (https://genome.jgi.doe.gov/portal/IMG_VR/IMG_VR.home.html). Source data are provided with this paper. All related supplementary files and Source Data file are available in https://github.com/ChenTianYi99/MR-SNWDC_virus.

## References

[1] Weitz JS, Beckett SJ, Brum JR, Cael BB, Dushoff J (2017). Lysis, lysogeny and virus–microbe ratios. Nature..

[2] Knowles B, Silveira CB, Bailey BA, Barott K, Cantu VA, Cobián-Güemes AG (2016). Lytic to temperate switching of viral communities. Nature..

[3] Howard-Varona C, Hargreaves KR, Abedon ST, Sullivan MB (2017). Lysogeny in nature: mechanisms, impact and ecology of temperate phages. ISME J..

[4] Alarcón-Schumacher T, Guajardo-Leiva S, Martinez-Garcia M, Díez B, Bernstein HC (2021). Ecogenomics and adaptation strategies of southern ocean viral communities. mSystems..

[5] Mooshammer M, Hofhansl F, Frank AH, Wanek W, Hämmerle I, Leitner S (2017). Decoupling of microbial carbon, nitrogen, and phosphorus cycling in response to extreme temperature events. Sci Adv.

[6] Pourtois J, Tarnita CE, Bonachela JA Impact of lytic phages on phosphorus- vs. nitrogen-limited marine microbes. Front Microbiol. 2020. 10.3389/fmicb.2020.00221. 10.3389/fmicb.2020.00221 PMC7047511 32153528

[7] Dodds WK, Jones JR, Welch EB (1998). Suggested classification of stream trophic state: distributions of temperate stream types by chlorophyll, total nitrogen, and phosphorus. Water Res.

[8] Tong Y, Qiao Z, Wang X, Liu X, Chen G, Zhang W (2018). Human activities altered water N:P ratios in the populated regions of China. Chemosphere..

[9] Pahlow M, Riebesell U (2000). Temporal trends in deep ocean Redfield ratios. Science..

[10] Krom MD, Kress N, Brenner S, Gordon LI (1991). Phosphorus limitation of primary productivity in the eastern Mediterranean Sea. Limnol Oceanogr.

[11] Karl D, Letelier R, Tupas L, Dore J, Christian J, Hebel D (1997). The role of nitrogen fixation in biogeochemical cycling in the subtropical North Pacific Ocean. Nature..

[12] Martiny AC, Pham CTA, Primeau FW, Vrugt JA, Moore JK, Levin SA (2013). Strong latitudinal patterns in the elemental ratios of marine plankton and organic matter. Nat Geosci.

[13] Schanz F, Juon H (1983). Two different methods of evaluating nutrient limitations of periphyton bioassays, using water from the River Rhine and eight of its tributaries. Hydrobiologia..

[14] Guildford SJ, Hecky RE (2000). Total nitrogen, total phosphorus, and nutrient limitation in lakes and oceans: Is there a common relationship?. Limnol Oceanogr.

[15] Liang Z, Soranno PA, Wagner T (2020). The role of phosphorus and nitrogen on chlorophyll a: evidence from hundreds of lakes. Water Res.

[16] Kim D, Lim J-H, Chun Y, Nayna OK, Begum MS, Park J-H (2021). Phytoplankton nutrient use and CO_2_ dynamics responding to long-term changes in riverine N and P availability. Water Res.

[17] Liu T, Zhang AN, Wang JW, Liu SF, Jiang XT, Dang CY (2018). Integrated biogeography of planktonic and sedimentary bacterial communities in the Yangtze River. Microbiome..

[18] Viaroli P, Soana E, Pecora S, Laini A, Naldi M, Fano EA (2018). Space and time variations of watershed N and P budgets and their relationships with reactive N and P loadings in a heavily impacted river basin (Po river, Northern Italy). Sci Total Environ.

[19] Lockwood S, Greening C, Baltar F, Morales SE (2022). Global and seasonal variation of marine phosphonate metabolism. ISME J.

[20] Shi H, Westfall CS, Kao J, Odermatt PD, Anderson SE, Cesar S (2021). Starvation induces shrinkage of the bacterial cytoplasm. Proc Natl Acad Sci USA.

[21] Tetu SG, Brahamsha B, Johnson DA, Tai V, Phillippy K, Palenik B (2009). Microarray analysis of phosphate regulation in the marine *Cyanobacterium Synechococcus* sp. WH8102. ISME J..

[22] Berg M, Goudeau D, Olmsted C, McMahon KD, Yitbarek S, Thweatt JL (2021). Host population diversity as a driver of viral infection cycle in wild populations of green sulfur bacteria with long standing virus-host interactions. ISME J.

[23] Rodriguez-Valera F, Martin-Cuadrado A-B, Rodriguez-Brito B, Pašić L, Thingstad TF, Rohwer F (2009). Explaining microbial population genomics through phage predation. Nat Rev Microbiol.

[24] Touchon M, Bernheim A, Rocha EPC (2016). Genetic and life-history traits associated with the distribution of prophages in bacteria. ISME J.

[25] Silveira CB, Rohwer FL (2016). Piggyback-the-Winner in host-associated microbial communities. npj Biofilms Microbi.

[26] Reyes A, Haynes M, Hanson N, Angly FE, Heath AC, Rohwer F (2010). Viruses in the faecal microbiota of monozygotic twins and their mothers. Nature..

[27] Williamson KE, Radosevich M, Smith DW, Wommack KE (2007). Incidence of lysogeny within temperate and extreme soil environments. Environ Microbiol.

[28] Paul JH (2008). Prophages in marine bacteria: dangerous molecular time bombs or the key to survival in the seas?. ISME J.

[29] Zablocki O, Adriaenssens EM, Cowan D (2016). Diversity and ecology of viruses in hyperarid desert soils. Appl Environ Microbiol.

[30] Kieft K, Zhou Z, Anderson RE, Buchan A, Campbell BJ, Hallam SJ (2021). Ecology of inorganic sulfur auxiliary metabolism in widespread bacteriophages. Nat Commun.

[31] Sullivan MB, Huang KH, Ignacio-Espinoza JC, Berlin AM, Kelly L, Weigele PR (2010). Genomic analysis of oceanic cyanobacterial myoviruses compared with T4-like myoviruses from diverse hosts and environments. Environ Microbiol.

[32] Benami M, Gillor O, Gross A (2016). Potential microbial hazards from graywater reuse and associated matrices: a review. Water Res.

[33] Yee RA, Leifels M, Scott C, Ashbolt NJ, Liu Y (2019). Evaluating microbial and chemical hazards in commercial struvite recovered from wastewater. Environ Sci Technol.

[34] Eichler S, Christen R, Höltje C, Westphal P, Bötel J, Brettar I (2006). Composition and dynamics of bacterial communities of a drinking water supply system as assessed by RNA- and DNA-based 16S rRNA gene fingerprinting. Appl Environ Microbiol.

[35] Frey KG, Herrera-Galeano JE, Redden CL, Luu TV, Servetas SL, Mateczun AJ (2014). Comparison of three next-generation sequencing platforms for metagenomic sequencing and identification of pathogens in blood. BMC Genomics.

[36] Wang JW, Pan R, Dong PY, Liu SF, Chen Q, Borthwick AGL (2022). Supercarriers of antibiotic resistome in a world’s large river. Microbiome..

[37] Zhang M, He L-Y, Liu Y-S, Zhao J-L, Zhang J-N, Chen J (2020). Variation of antibiotic resistome during commercial livestock manure composting. Environ Int.

[38] Tyson GW, Chapman J, Hugenholtz P, Allen EE, Ram RJ, Richardson PM (2004). Community structure and metabolism through reconstruction of microbial genomes from the environment. Nature..

[39] Dong Z, Yan Y, Duan J, Fu X, Zhou Q, Huang X (2011). Computing payment for ecosystem services in watersheds: an analysis of the Middle Route Project of South-to-North Water Diversion in China. J Environ Sci.

[40] Zhang L, Yin W, Wang C, Zhang A, Zhang H, Zhang T (2021). Untangling microbiota diversity and assembly patterns in the world’s largest water diversion canal. Water Res.

[41] Chen W, Ren K, Isabwe A, Chen H, Liu M, Yang J (2019). Stochastic processes shape microeukaryotic community assembly in a subtropical river across wet and dry seasons. Microbiome..

[42] Huang J, Zhang Y, Bing H, Peng J, Dong F, Gao J (2021). Characterizing the river water quality in China: Recent progress and on-going challenges. Water Res.

[43] Wang J, Liu Q, Zhao X, Borthwick AGL, Liu Y, Chen Q (2019). Molecular biogeography of planktonic and benthic diatoms in the Yangtze River. Microbiome..

[44] Wang J, Liu T, Sun W, Chen Q (2020). Bioavailable metal(loid)s and physicochemical features co-mediating microbial communities at combined metal(loid) pollution sites. Chemosphere..

[45] Uritskiy GV, DiRuggiero J, Taylor J (2018). MetaWRAP—a flexible pipeline for genome-resolved metagenomic data analysis. Microbiome..

[46] Li D, Liu C-M, Luo R, Sadakane K, Lam T-W (2015). MEGAHIT: an ultra-fast single-node solution for large and complex metagenomics assembly via succinct *de Bruijn* graph. Bioinformatics..

[47] Bowers RM, Kyrpides NC, Stepanauskas R, Harmon-Smith M, Doud D, Reddy TBK (2017). Minimum information about a single amplified genome (MISAG) and a metagenome-assembled genome (MIMAG) of bacteria and archaea. Nat Biotechnol.

[48] Chaumeil PA, Mussig AJ, Hugenholtz P, Parks DH (2019). GTDB-Tk: a toolkit to classify genomes with the Genome Taxonomy Database. Bioinformatics..

[49] Antipov D, Raiko M, Lapidus A, Pevzner PA (2020). MetaviralSPAdes: assembly of viruses from metagenomic data. Bioinformatics.

[50] Kieft K, Zhou ZC, Anantharaman K (2020). VIBRANT: automated recovery, annotation and curation of microbial viruses, and evaluation of viral community function from genomic sequences. Microbiome..

[51] Ren J, Song K, Deng C, Ahlgren NA, Fuhrman JA, Li Y (2020). Identifying viruses from metagenomic data using deep learning. Quant Biol.

[52] Fang ZC, Tan J, Wu SF, Li M, Xu CM, Xie ZJ (2019). PPR-Meta: a tool for identifying phages and plasmids from metagenomic fragments using deep learning. Gigascience..

[53] Guo J, Bolduc B, Zayed AA, Varsani A, Dominguez-Huerta G, Delmont TO (2021). VirSorter2: a multi-classifier, expert-guided approach to detect diverse DNA and RNA viruses. Microbiome..

[54] Nayfach S, Camargo AP, Schulz F, Eloe-Fadrosh E, Roux S, Kyrpides NC (2021). CheckV assesses the quality and completeness of metagenome-assembled viral genomes. Nat Biotechnol.

[55] Fu LM, Niu BF, Zhu ZW, Wu ST, Li WZ (2012). CD-HIT: accelerated for clustering the next-generation sequencing data. Bioinformatics..

[56] Hockenberry AJ, Wilke CO (2021). BACPHLIP: predicting bacteriophage lifestyle from conserved protein domains. PeerJ..

[57] Li Z, Pan D, Wei G, Pi W, Zhang C, Wang J-H (2021). Deep sea sediments associated with cold seeps are a subsurface reservoir of viral diversity. ISME J.

[58] Tominaga K, Morimoto D, Nishimura Y, Ogata H, Yoshida T *In silico* prediction of virus-host interactions for marine bacteroidetes with the use of metagenome-assembled genomes. Front Microbiol. 2020. 10.3389/fmicb.2020.00738. 10.3389/fmicb.2020.00738 PMC7198788 32411107

[59] Dutilh BE, Cassman N, McNair K, Sanchez SE, Silva GGZ, Boling L (2014). A highly abundant bacteriophage discovered in the unknown sequences of human faecal metagenomes. Nat Commun.

[60] Gouy M, Gautier C (1982). Codon usage in bacteria: correlation with gene expressivity. Nucleic Acids Res.

[61] Laslett D, Canback B (2004). ARAGORN, a program to detect tRNA genes and tmRNA genes in nucleotide sequences. Nucleic Acids Res.

[62] Horvath P, Barrangou R (2010). CRISPR/Cas, the immune system of bacteria and archaea. Science.

[63] Bland C, Ramsey TL, Sabree F, Lowe M, Brown K, Kyrpides NC (2007). CRISPR Recognition Tool (CRT): a tool for automatic detection of clustered regularly interspaced palindromic repeats. BMC Bioinform.

[64] Emerson JB, Roux S, Brum JR, Bolduc B, Woodcroft BJ, Jang HB (2018). Host-linked soil viral ecology along a permafrost thaw gradient. Nat Microbiol.

[65] Yin X, Jiang XT, Chai B, Li L, Yang Y, Cole JR (2018). ARGs-OAP v2.0 with an expanded SARG database and Hidden Markov Models for enhancement characterization and quantification of antibiotic resistance genes in environmental metagenomes. Bioinformatics..

[66] Liu B, Zheng D, Zhou S, Chen L, Yang J (2021). VFDB 2022: a general classification scheme for bacterial virulence factors. Nucleic Acids Res.

[67] Lee K, Kim D-W, Lee D-H, Kim Y-S, Bu J-H, Cha J-H (2020). Mobile resistome of human gut and pathogen drives anthropogenic bloom of antibiotic resistance. Microbiome..

[68] Huerta-Cepas J, Forslund K, Coelho LP, Szklarczyk D, Jensen LJ, von Mering C (2017). Fast genome-wide functional annotation through orthology assignment by eggNOG-mapper. Mol Biol Evol.

[69] Huerta-Cepas J, Szklarczyk D, Heller D, Hernández-Plaza A, Forslund SK, Cook H (2018). eggNOG 5.0: a hierarchical, functionally and phylogenetically annotated orthology resource based on 5090 organisms and 2502 viruses. Nucleic Acids Res.

[70] Mende DR, Sunagawa S, Zeller G, Bork P (2013). Accurate and universal delineation of prokaryotic species. Nat Methods.

[71] Mende DR, Bryant JA, Aylward FO, Eppley JM, Nielsen T, Karl DM (2017). Environmental drivers of a microbial genomic transition zone in the ocean’s interior. Nat Microbiol.

[72] Roux S, Páez-Espino D, Chen I-MA, Palaniappan K, Ratner A, Chu K (2020). IMG/VR v3: an integrated ecological and evolutionary framework for interrogating genomes of uncultivated viruses. Nucleic Acids Res.

[73] Griffith DA, Peres-Neto PR (2006). Spatial modeling in ecology: the flexibility of eigenfunction spatial analyses. Ecology.

[74] Breiman L (2001). Random forests. Mach Learn.

[75] Jiao S, Chen W, Wang J, Du N, Li Q, Wei G (2018). Soil microbiomes with distinct assemblies through vertical soil profiles drive the cycling of multiple nutrients in reforested ecosystems. Microbiome..

[76] Röttjers L, Faust K (2018). From hairballs to hypotheses–biological insights from microbial networks. FEMS Microbiol Rev.

[77] Zhang B, Zhang J, Liu Y, Shi P, Wei G (2018). Co-occurrence patterns of soybean rhizosphere microbiome at a continental scale. Soil Biol Biochem.

[78] Gregory AC, Zayed AA, Conceição-Neto N, Temperton B, Bolduc B, Alberti A (2019). Marine DNA viral macro- and microdiversity from pole to pole. Cell.

[79] Li Z, Zhang R, Liu C, Zhang R, Chen F, Liu Y (2020). Phosphorus spatial distribution and pollution risk assessment in agricultural soil around the Danjiangkou reservoir, China. Sci Total Environ.

[80] Reis PCJ, Thottathil SD, Prairie YT (2022). The role of methanotrophy in the microbial carbon metabolism of temperate lakes. Nat Commun.

[81] Siddiqui KS, Poljak A, Guilhaus M, De Francisci D, Curmi PMG, Feller G (2006). Role of lysine versus arginine in enzyme cold-adaptation: modifying lysine to homo-arginine stabilizes the cold-adapted α-amylase from *Pseudoalteramonas haloplanktis*. Proteins..

[82] Kelly L, Ding H, Huang KH, Osburne MS, Chisholm SW (2013). Genetic diversity in cultured and wild marine cyanomyoviruses reveals phosphorus stress as a strong selective agent. ISME J.

[83] Pasek MA, Harnmeijer JP, Buick R, Gull M, Atlas Z (2013). Evidence for reactive reduced phosphorus species in the early Archean ocean. Proc Natl Acad Sci USA.

[84] Lundin D, Torrents E, Poole AM, Sjöberg B-M (2009). RNRdb, a curated database of the universal enzyme family ribonucleotide reductase, reveals a high level of misannotation in sequences deposited to Genbank. BMC Genomics.

[85] Gon S, Camara JE, Klungsøyr HK, Crooke E, Skarstad K, Beckwith J (2006). A novel regulatory mechanism couples deoxyribonucleotide synthesis and DNA replication in *Escherichia coli*. EMBO J..

[86] Torrents E. Ribonucleotide reductases: essential enzymes for bacterial life. Front Cell Infect Microbiol. 2014. 10.3389/fcimb.2014.00052. 10.3389/fcimb.2014.00052 PMC4009431 24809024

[87] Zhou B, Su L, Hu S, Hu W, Yip MLR, Wu J (2013). A small-molecule blocking ribonucleotide reductase holoenzyme formation inhibits cancer cell growth and overcomes drug resistance. Cancer Res.

[88] Stincone A, Prigione A, Cramer T, Wamelink MMC, Campbell K, Cheung E (2015). The return of metabolism: biochemistry and physiology of the pentose phosphate pathway. Biol Rev.

[89] Sastre DE, Pulschen AA, Basso LGM, Benites Pariente JS, Marques Netto CGC, Machinandiarena F (2020). The phosphatidic acid pathway enzyme PlsX plays both catalytic and channeling roles in bacterial phospholipid synthesis. J Biol Chem.

[90] Cloutier P, Coulombe B (2013). Regulation of molecular chaperones through post-translational modifications: decrypting the chaperone code. Biochim Biophys Acta.

[91] Lindell D, Jaffe JD, Coleman ML, Futschik ME, Axmann IM, Rector T (2007). Genome-wide expression dynamics of a marine virus and host reveal features of co-evolution. Nature..

[92] Suttle CA (2007). Marine viruses — major players in the global ecosystem. Nat Rev Microbiol.

[93] Jover LF, Effler TC, Buchan A, Wilhelm SW, Weitz JS (2014). The elemental composition of virus particles: implications for marine biogeochemical cycles. Nat Rev Microbiol.

[94] Dyhrman ST, Ammerman JW, Van, Mooy BAS (2007). Microbes and the marine phosphorus cycle. Oceanography.

[95] Maat DS, van Bleijswijk JDL, Witte HJ, Brussaard CPD Virus production in phosphorus-limited *Micromonas pusilla* stimulated by a supply of naturally low concentrations of different phosphorus sources, far into the lytic cycle. FEMS Microbiol Ecol. 2016. 10.1093/femsec/fiw136. 10.1093/femsec/fiw136 27316561

[96] Breitbart M, Bonnain C, Malki K, Sawaya NA (2018). Phage puppet masters of the marine microbial realm. Nat Microbiol.

[97] Jones KE, Patel NG, Levy MA, Storeygard A, Balk D, Gittleman JL (2008). Global trends in emerging infectious diseases. Nature.

[98] Rehman S, Ali Z, Khan M, Bostan N, Naseem S (2019). The dawn of phage therapy. Rev Med Virol.

[99] Debarbieux L, Leduc D, Maura D, Morello E, Criscuolo A, Grossi O (2010). Bacteriophages can treat and prevent *Pseudomonas aeruginosa* lung infections. J Infect Dis.

[100] Lomelí-Ortega CO, Martínez-Díaz SF (2014). Phage therapy against *Vibrio parahaemolyticus* infection in the whiteleg shrimp (*Litopenaeus vannamei*) larvae. Aquaculture..

