# Supplementary material for: Virus–pathogen interactions improve water quality along the Middle Route of the South-to-North Water Diversion Canal
Source: ISME J. 2023 Jul 31;17(10):1719–32. doi: 10.1038/s41396-023-01481-2 (PMC10504254; doi:10.1038/s41396-023-01481-2)
Supplement: Supplementary file 1 — Supplementary Information [file 41396_2023_1481_MOESM1_ESM.pdf]

Supporting Information for

Virus–pathogen interactions improve water quality along the Middle Route of the South-to-North Water Diversion Canal

Tianyi Chen, Tang Liu, Zongzhi Wu, Bingxue Wang, Qian Chen, Mi Zhang, Enhang Liang and Jinren Ni*

*Jinren Ni, College of Environmental Sciences and Engineering, Peking University, Beijing 100871, P. R. China

E-mail: jinrenni@pku.edu.cn (J.R. Ni)

Supplemental Methods

Construction of virus–host infection network

The virus–host linkages were visualized by ForceAtlas 2 algorithm in Gephi v0.9.2 [1]. Modularity quantification was conducted using community detection method [2]. The feature set of the virus–host infection network included average degree and modularity index, which represented the number of adjacent edges and the connectivity of community members [3, 4].

Identification of auxiliary metabolic genes

Removal of host contamination and identification of prophage boundaries were performed via CheckV [5] in advance. The predicted protein sequences, generated by Prodigal [6], were aligned to the eggNOG database [7] using emapper.py v1.0.3 [8] (-m diamond; --seed_orthology_evalue 1e^{-5}). Each protein was assigned a COG annotation. AMG identification was first conducted by VIBRANT [9] according to KEGG, Pfam, and VOG databases. The genes with annotations “metabolic pathways” and “sulfur relay system” were regarded as putative AMGs. VirSorter2 [10] provided the information of virus-associated and viral hallmark genes within contigs, and generated annotation files for

DRAM-v [11] to perform the parallel AMG identification. The genes with M/F flag assignments and auxiliary scores of ≤ 3 were regarded as putative AMGs. In order to avoid false positive results, only the AMGs located between two virus-associated or viral hallmark genes and those located alongside the viral-associated or viral hallmark genes were selected for further analysis [12]. Phyre2 [13] was applied to identify tertiary protein structures with confidence > 90% and coverage > 70%. PROSITE [14] was used to analyze conserved regions and active sites of putative AMGs based on PROSITE collection of motifs. Genome maps for AMG-containing viral contigs were visualized based on COG, VIBRANT, VirSorter2, and DRAM-v annotations.

Comparisons of viral sequences in the MR-SNWDC and other freshwater ecosystems

Viral contigs with over 90% completeness were selected from the freshwater sources in the IMG/VR database [15], for subsequent viral clustering analysis with vOTUs in the MR-SNWDC. Each reported viral sequence was assigned to a specific ecosystem subtype (lake, lentic, groundwater, sediment, wetlands, river, ice, creek, lotic, pond, and drinking water). The protein sequences retrieved from Prodigal v2.6.3 [6] were used for gene-sharing network analysis through vConTACT2 v0.9.19 [16]. Diamond [17] was applied to estimate the protein–protein similarity. Protein clusters were calculated by the Markov Cluster Algorithm (MCL), with the subsequent VC generation using ClusterONE [18].

Supplemental Results

Relationship between vOTUs in the MR-SNWDC and publicly reported viral sequences in the IMG/VR database

Gene-sharing network analysis was performed to evaluate the relationship

between 40,261 vOTUs in the MR-SNWDC and 37,364 viral sequences (>90% completeness) from a broader diversity of freshwater ecosystems in the IMG/VR database [15]. Around half of vOTUs in the MR-SNWDC were assigned to 7,389 viral clusters (VCs) at the genus level, with 68.2% VCs not including viruses from any other ecosystems in the IMG/VR database (Fig. S10A). Only 9.1% of identified vOTUs were clustered with publicly reported viruses, suggesting that the MR-SNWDC was an endemic pool of diverse and novel freshwater viruses. Among 3,670 vOTUs which shared VCs with publicly available viruses, over 85% were clustered with viral sequences from the lake source. In addition, about one thirds of lake-derived viral genera were clustered with vOTUs in the MR-SNWDC, ranking the most among all freshwater sources (Fig. S10B), which highlighted the role of Danjiangkou Reservoir (lake-like) in shaping the viral communities across the canal.

Supplemental Figures

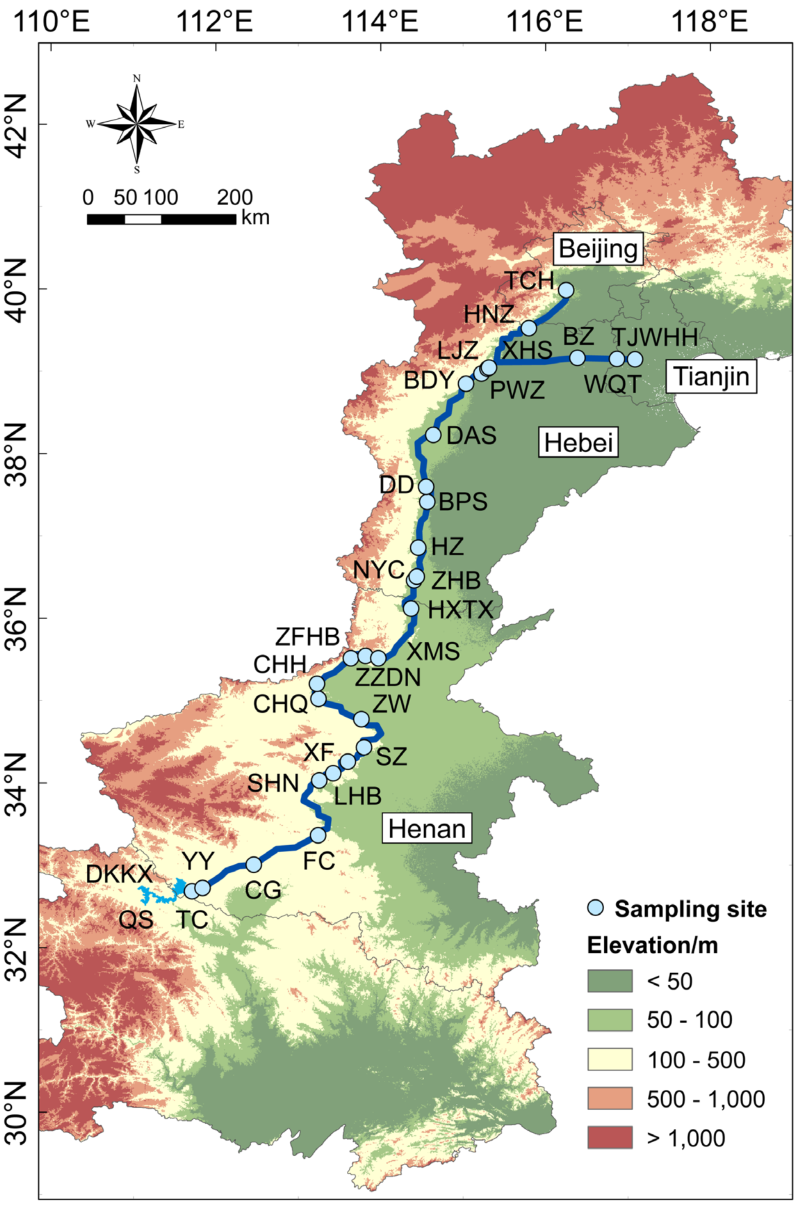

Fig. S1 Sketch map of the MR-SNWDC. Sampling sites are distributed at 32 monitoring stations along the water canal (see Table S1). The length of the canal (1,432 km) is measured by the sum of the dendritic distances between each pair of adjacent sampling sites, as an indication of canal network density, rather than the straight-line distance between the water source area and the canal end. Sampling campaigns are carried out at the same sites in August 2020 and March 2021, respectively.

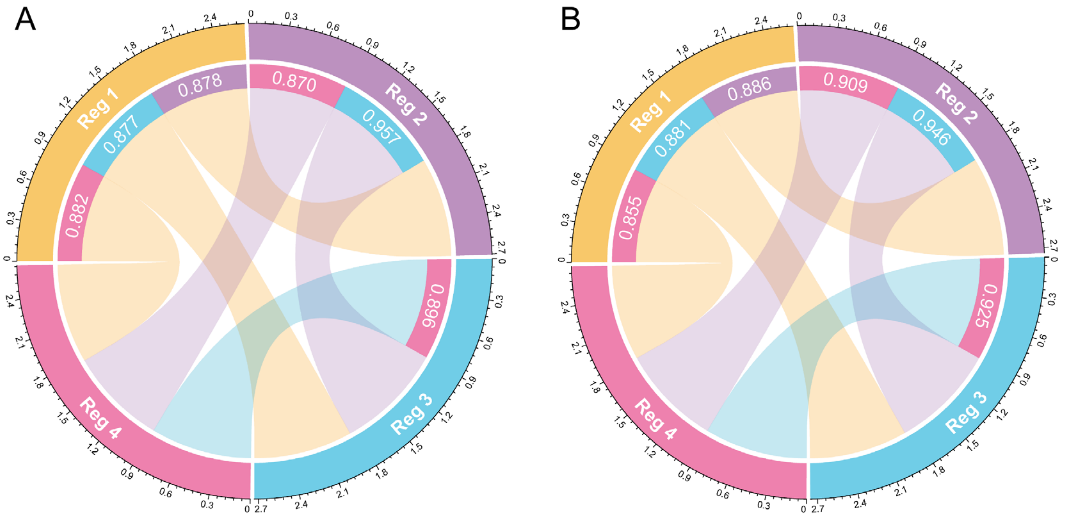

Fig. S2 Regional similarity of viral communities in autumn (A) and spring (B). Sorenson similarity is calculated for the relative abundances of vOTUs. The width of each curve represents the similarity value between the paired regions. Source data are provided in the Source Data file.

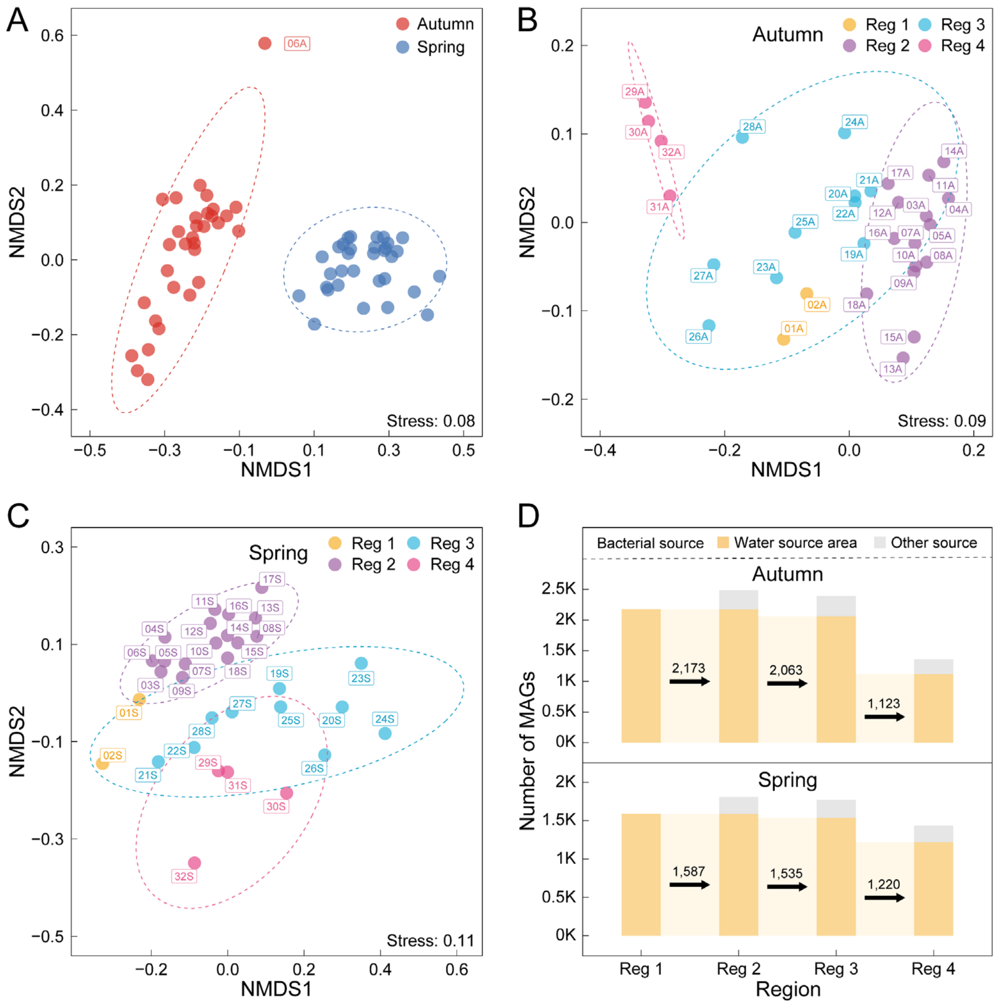

Fig. S3 Spatiotemporal distribution of bacterial communities in autumn and spring. Nonmetric multidimensional scaling (NMDS) analyses visualize the temporal variation of bacterial β-diversity (**A**) as well as the distinct partition of bacterial communities into four ecological regions in autumn (**B**) and spring (**C**), based on the Bray–Curtis dissimilarity matrix calculated from the relative abundances of prokaryotic MAGs. The stress value denotes the ordination fitness of each NMDS plot. Each group is encircled by an ellipse at 95% confidence interval. One outlier sample (06A) is excluded from subsequent analyses. **D** The richness of observed bacterial species transported from the water source area (Reg 1) to downstream regions (Reg 2~4) in autumn (upper panel) and spring (lower panel). Source data are provided in the Source Data file.

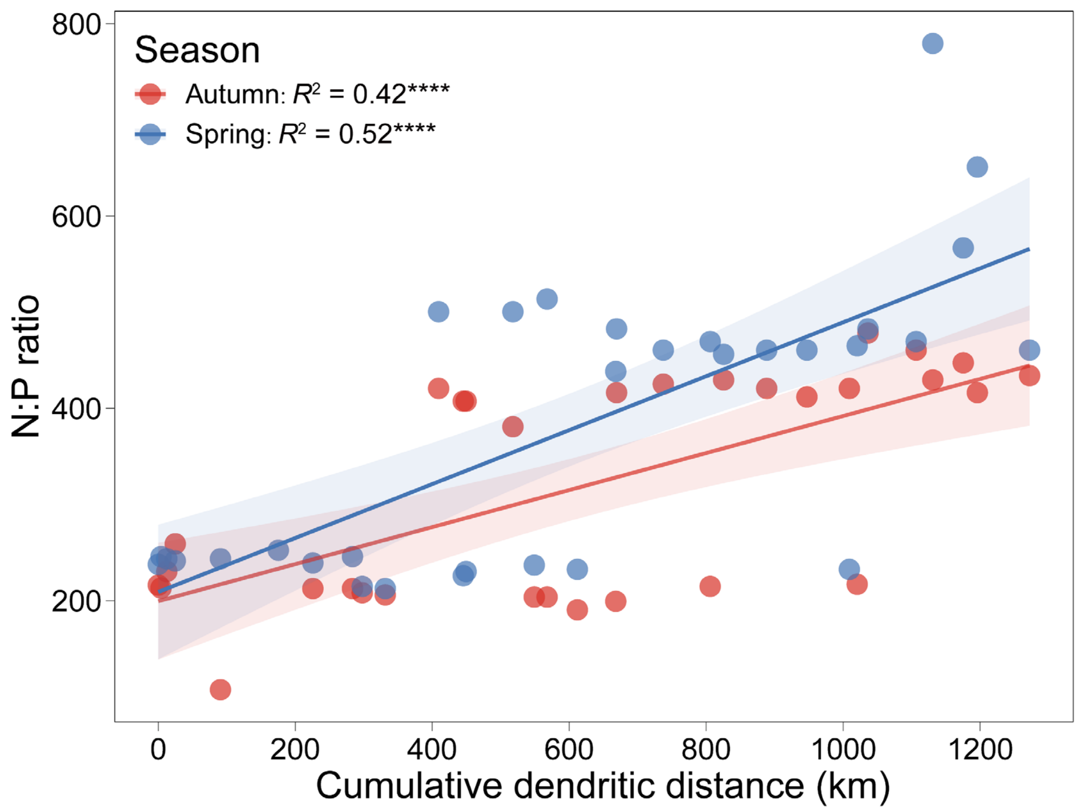

Fig. S4 Changes in the N:P ratio (molar) along the canal in autumn and spring. The goodness-of-fit R^2 value and the statistical significance are presented for each linear regression (****: < 0.0001). Source data are provided in the Source Data file.

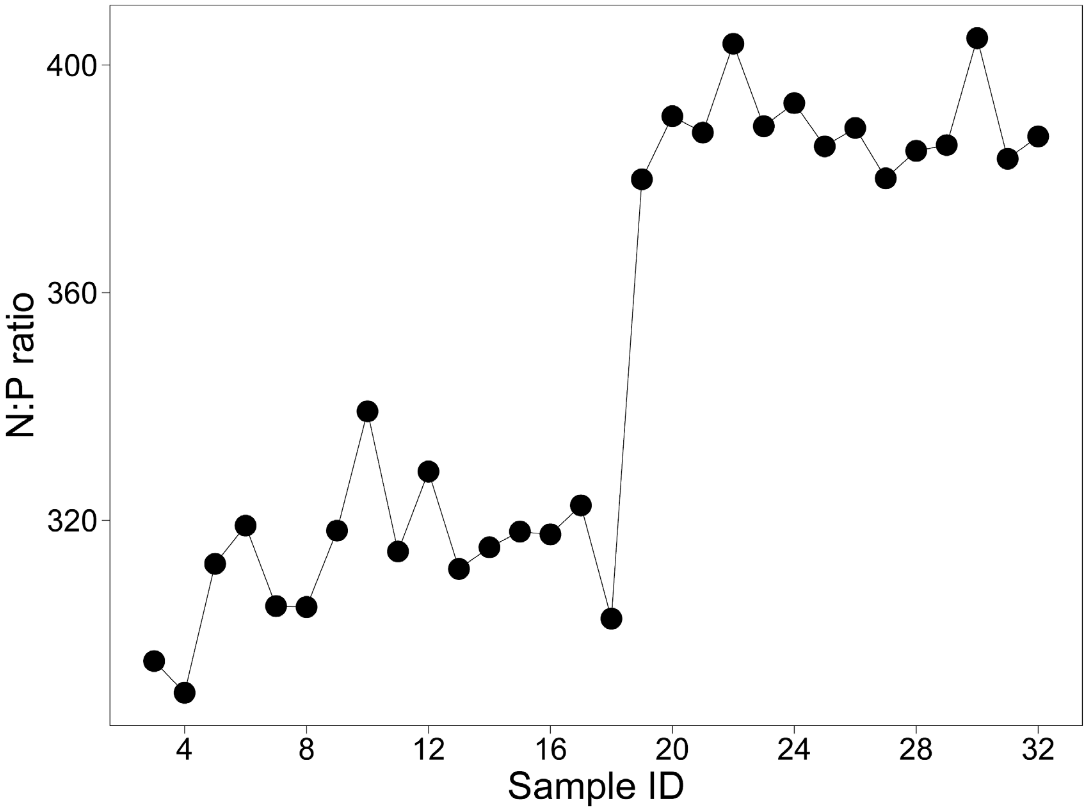

Fig. S5 Changes in the N:P ratio (molar) along the main canal during 2015~2021. Source data are provided in the Source Data file.

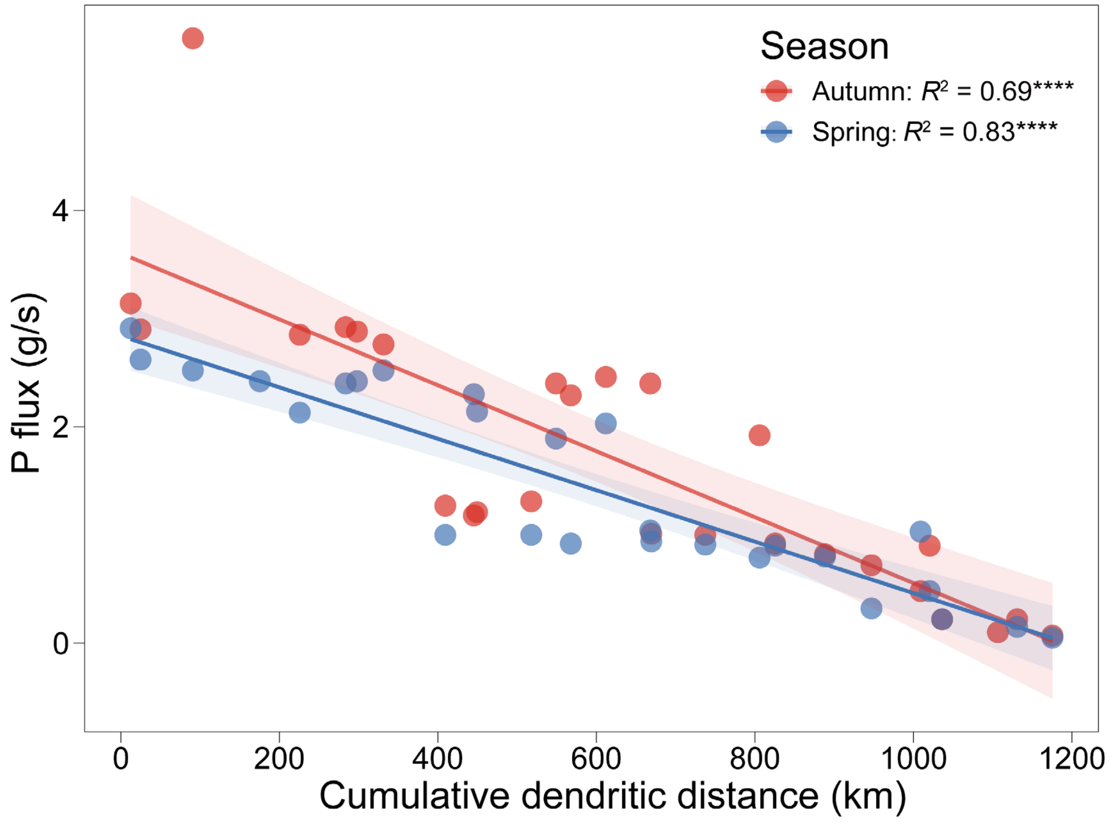

Fig. S6 Changes in P flux along the canal in autumn and spring (concentration × flow rate, g/s). Each linear regression is denoted by the goodness-of-fit R^2 value and the significance level of p value (****: < 0.0001). Source data are provided in the Source Data file.

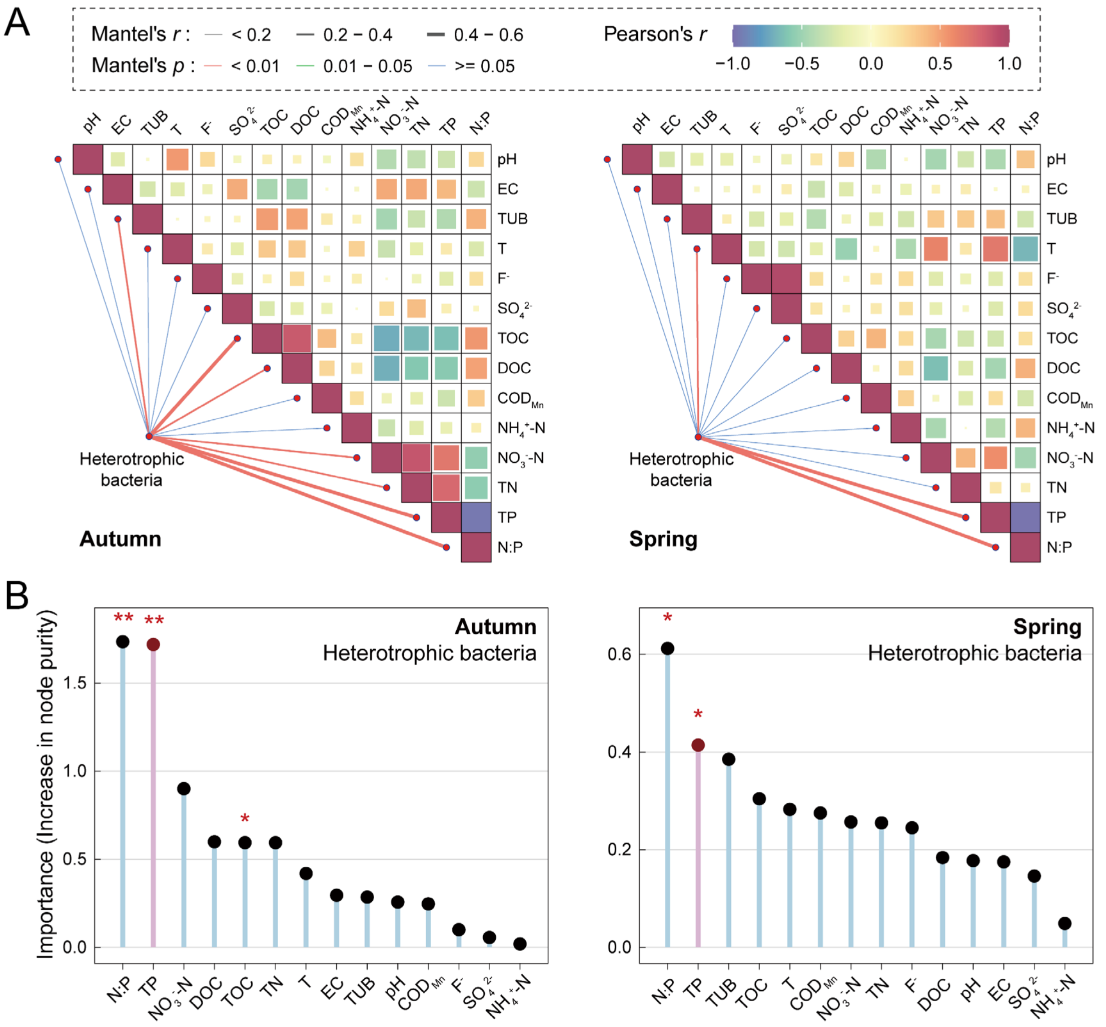

Fig. S7 Relevance of environmental factors and heterotrophic bacterial communities. A Correlation between environmental factors and heterotrophic bacterial communities in autumn and spring. Pairwise Pearson's coefficients are denoted by color gradients. Edge width demonstrates the Mantel's *r* correlation coefficients. Edge color represents the significance level of *p* value based on 999 permutations. **B** Random forest importance of each environmental factor for heterotrophic bacterial communities in two seasons. All environmental factors are brought into a ranking by their importance index represented by the increase in node purity. The significance of each environmental factor is shown in asterisks (**: < 0.01; *: < 0.05). Source data are provided in the Source Data file.

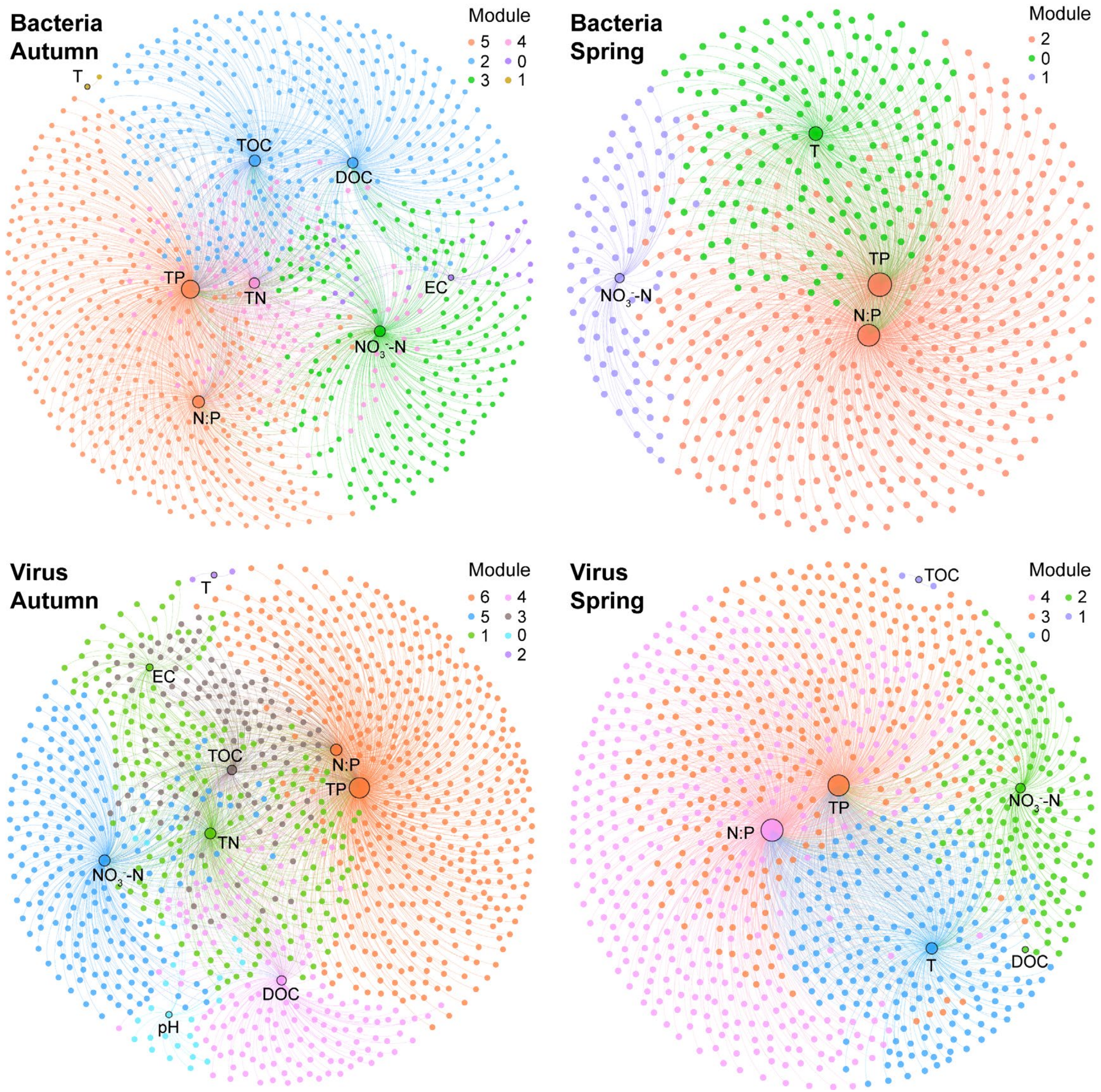

Fig. S8 Co-occurrence network of environmental factors and MAGs/vOTUs. The size of each dot marking environmental factors is proportional to the number of connections. Dots with different colors denote different modules in networks. Source data are provided in the Source Data file.

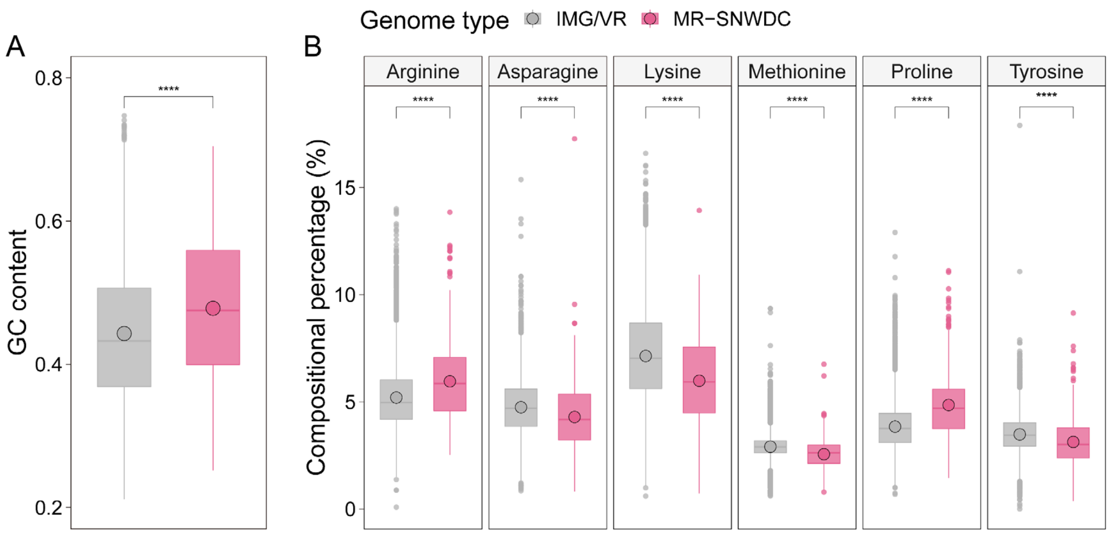

Fig. S9 Molecular properties of viral genomes in the MR-SNWDC and the IMG/VR database. Differences in GC content (**A**) and specific amino acid frequencies (**B**) are estimated by Bonferroni-adjusted Wilcoxon test. The statistical significance is marked by asterisks (****: ≤ 0.0001). Source data are provided in the Source Data file.

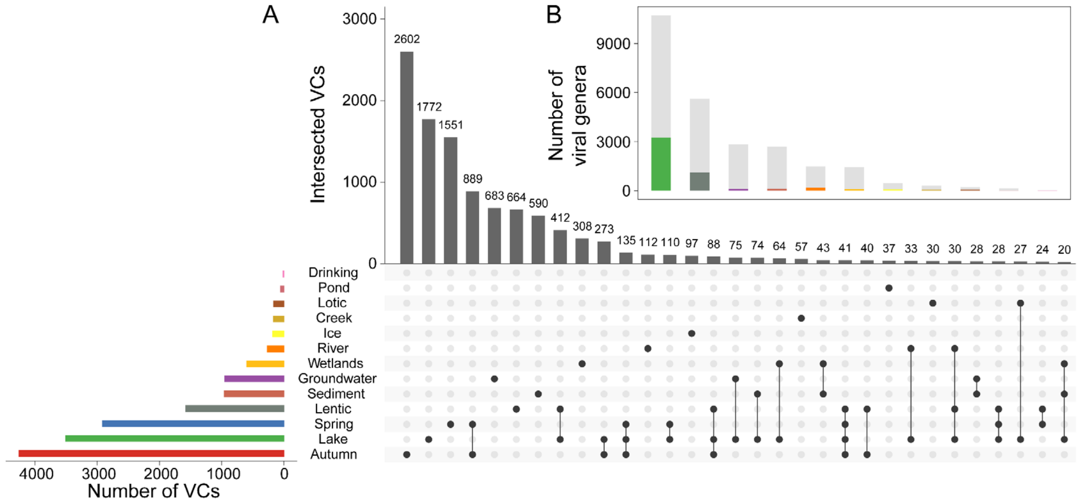

Fig. S10 Comparison of viral species in the MR-SNWDC and the IMG/VR database. A Shared viral clusters (VCs) among different datasets. Viral sequences from 11 freshwater ecosystems are selected from the IMG/VR database. Each source of VCs is defined as a set. The bars on the left represent the total number of VCs in each set. Dots with interconnecting vertical black lines represent the intersections, where black dots represent sets that were within the intersection and unfilled light gray dots represent sets that were not part of the intersection. The bars on the top right represent the number of VCs within the intersection. **B** Proportional number of viral genera from diverse freshwater sources in the IMG/VR database which are clustered with vOTUs in the MR-SNWDC. Source data are provided in the Source Data file.

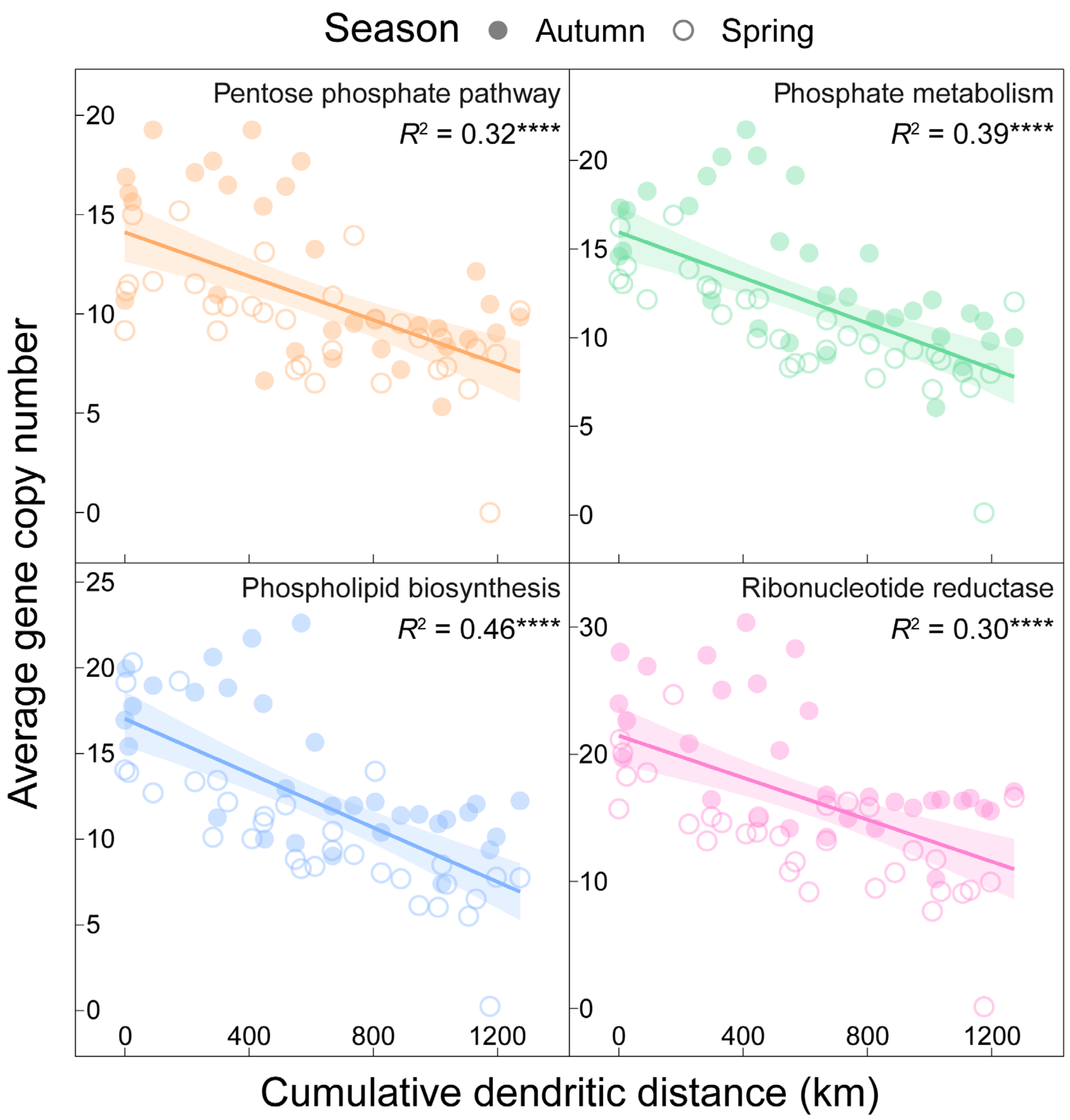

Fig. S11 Changes in average copy number of bacteria-encoded genes involved in key P-associated metabolic processes along the canal. The goodness-of-fit R^2 value and the significance level of p value are presented for each linear regression (****: ≤ 0.0001). Source data are provided in the Source Data file.

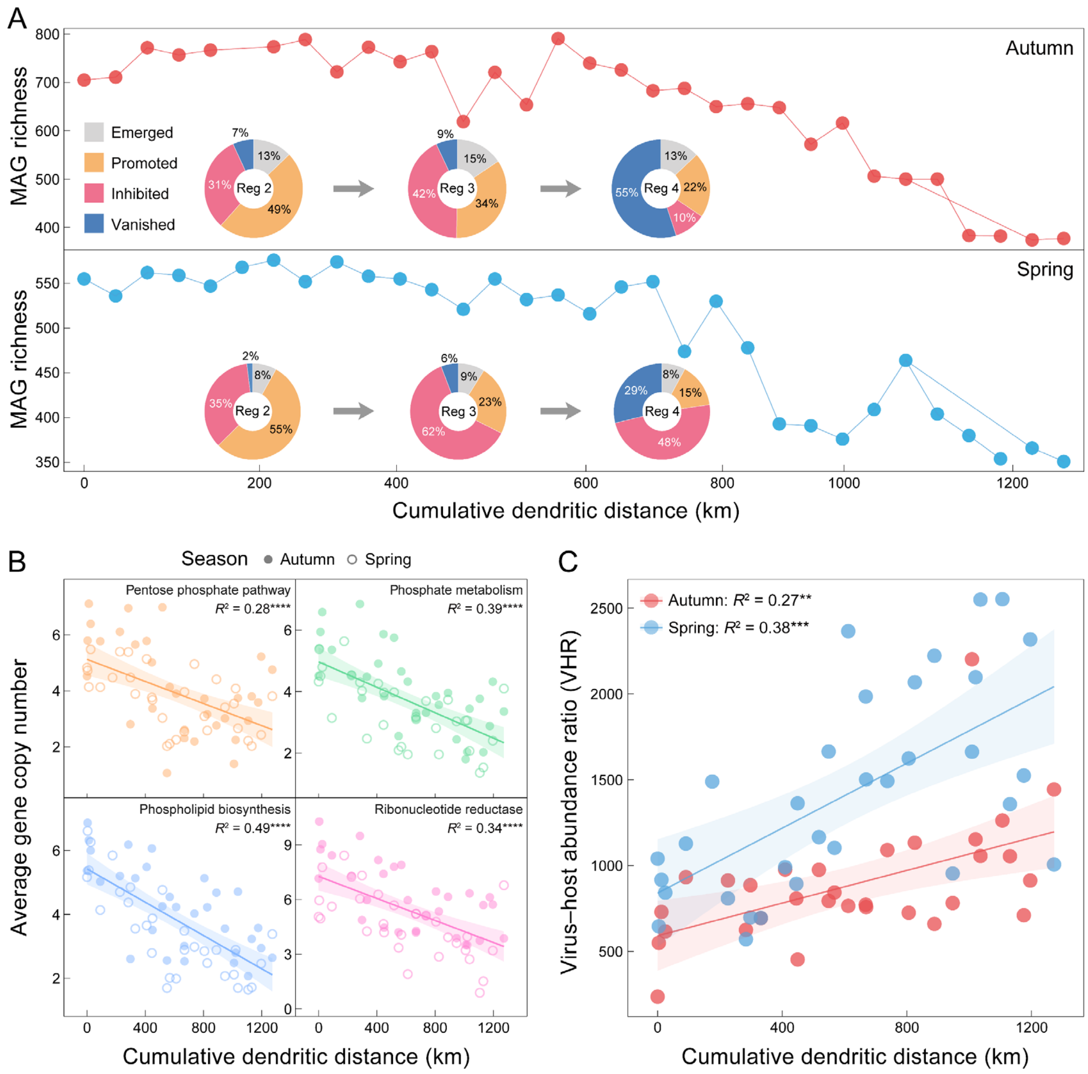

Fig. S12. Dynamics and P-associated functions of bacteria with high-quality genomes (completeness > 90%, contamination < 5%), as well as their relationships with viruses. A Changes in the richness (line charts) and growth potential (pie charts, see Materials and Methods) of bacteria along the canal in autumn and spring. **B** Changes in average copy number of key bacteria-encoded genes of four metabolic processes associated with P acquisition and utilization. **C** Virus–host abundance ratios display notable increase with water flow in both seasons. Each linear regression is denoted by the goodness-of-fit R^2 value and the significance level of *p* value. Source data are provided in the Source Data file.

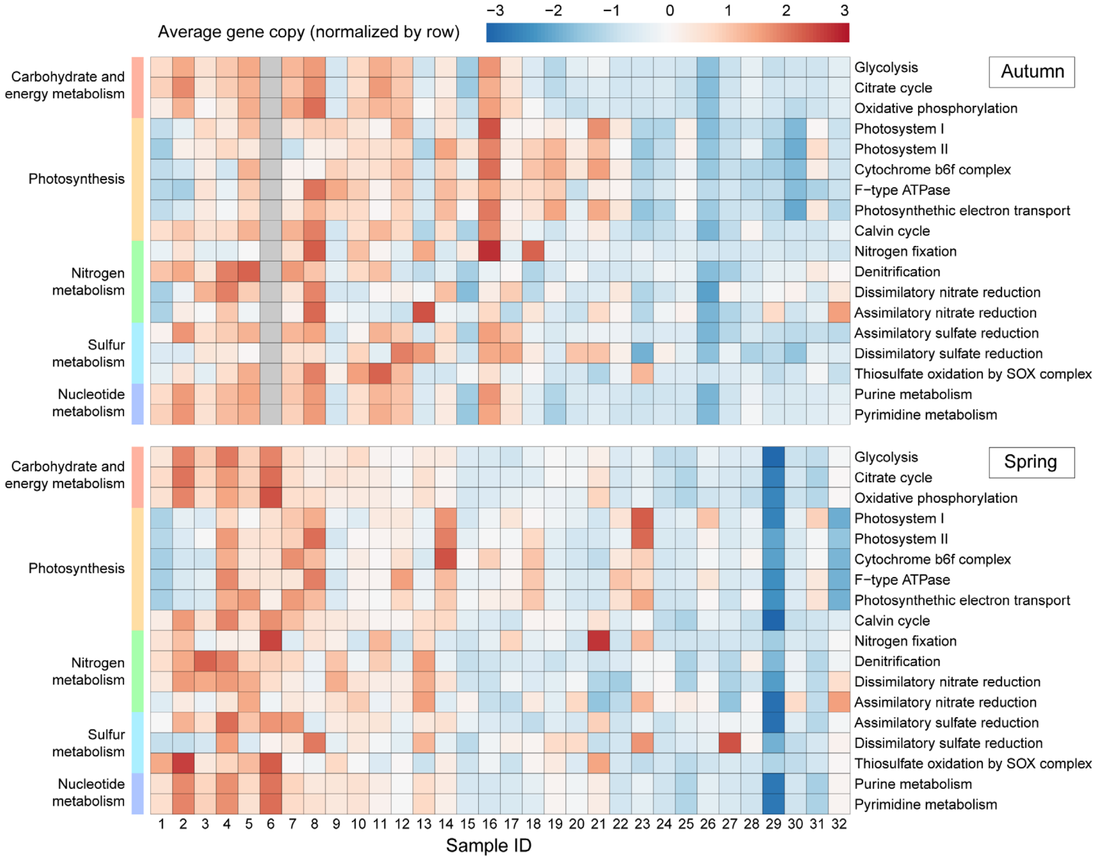

Fig. S13 Changes in average copy number of functional genes involved in carbohydrate, energy, nitrogen, sulfur, and nucleotide metabolism along the canal in autumn and spring. The average gene copy is normalized by each KEGG pathway/module. Source data are provided in the Source Data file.

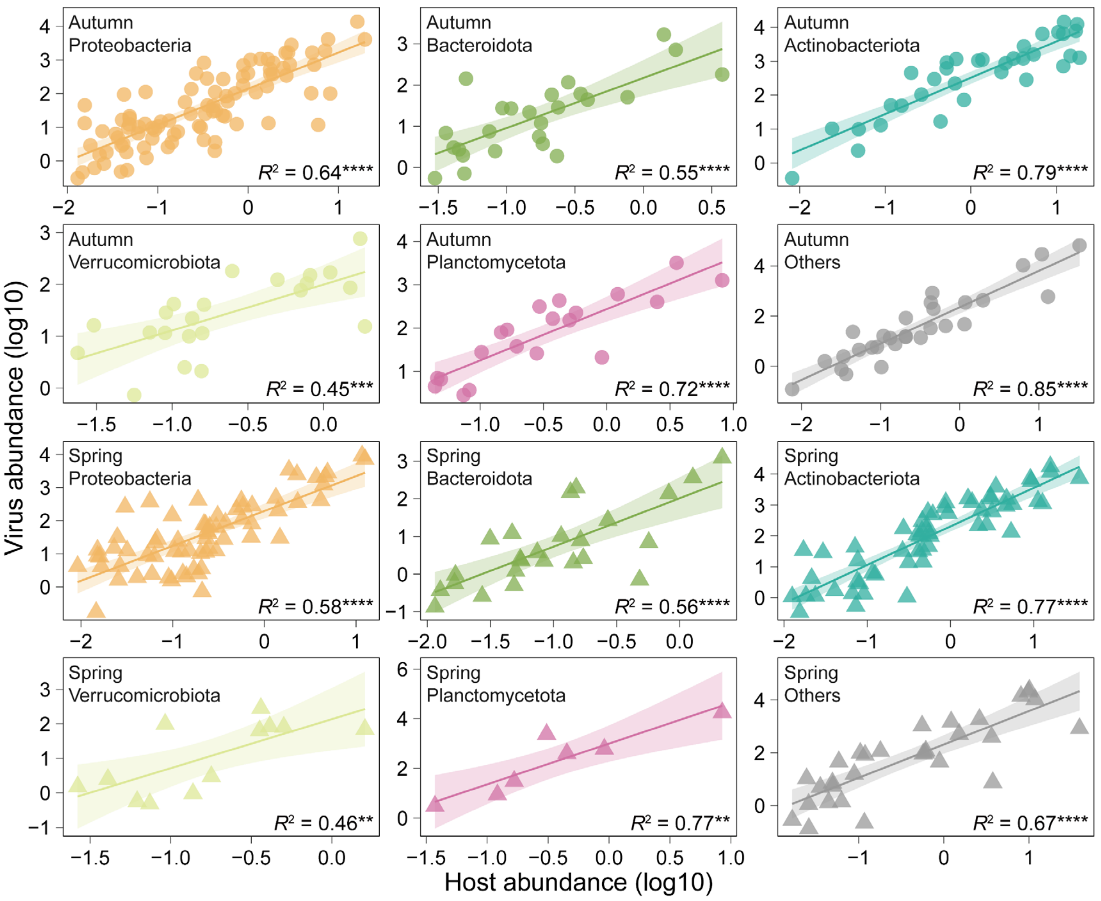

Fig. S14 Correlation between abundances of viruses and their hosts for each phylum in autumn and spring. Host phyla linked to relatively fewer viruses are categorized into “Others”. Each linear regression is denoted by the goodness-of-fit R^2 value and the significance level of p value. Source data are provided in the Source Data file.

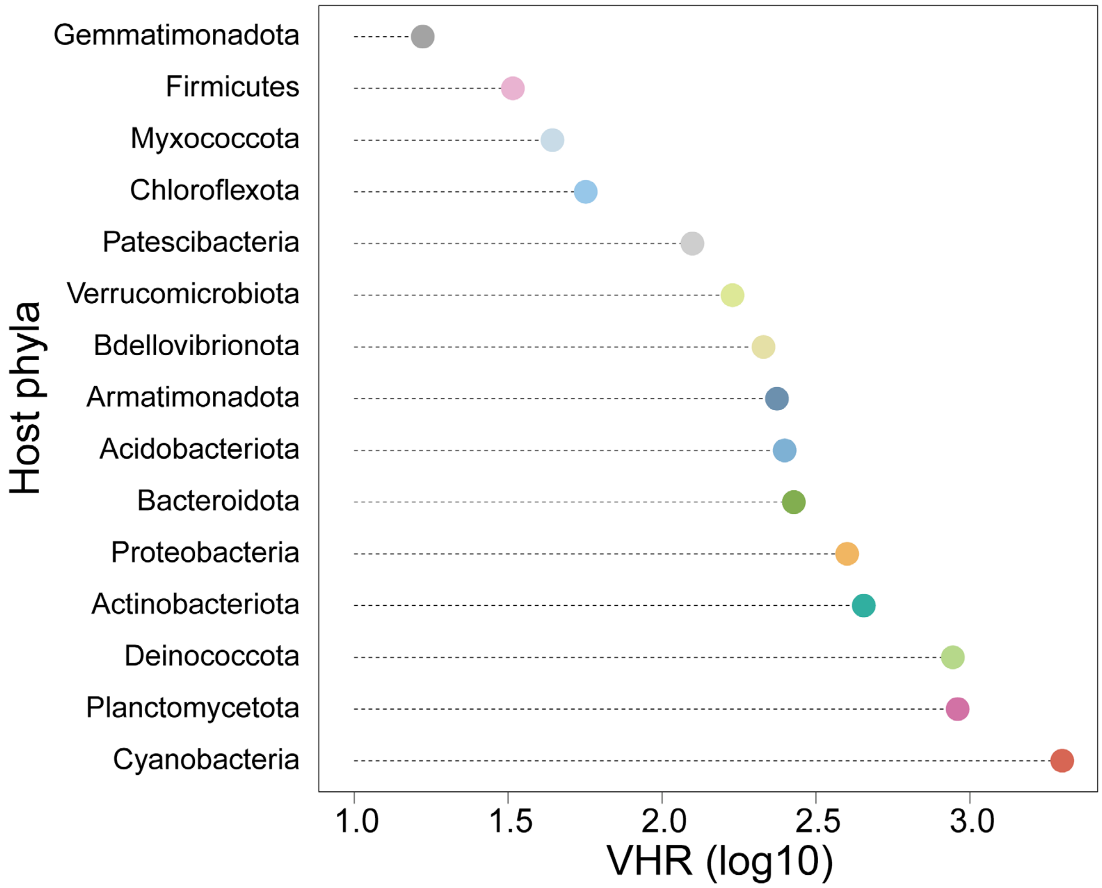

Fig. S15 Virus–host abundance ratios (VHR) for each bacterial phylum. Source data are provided in the Source Data file.

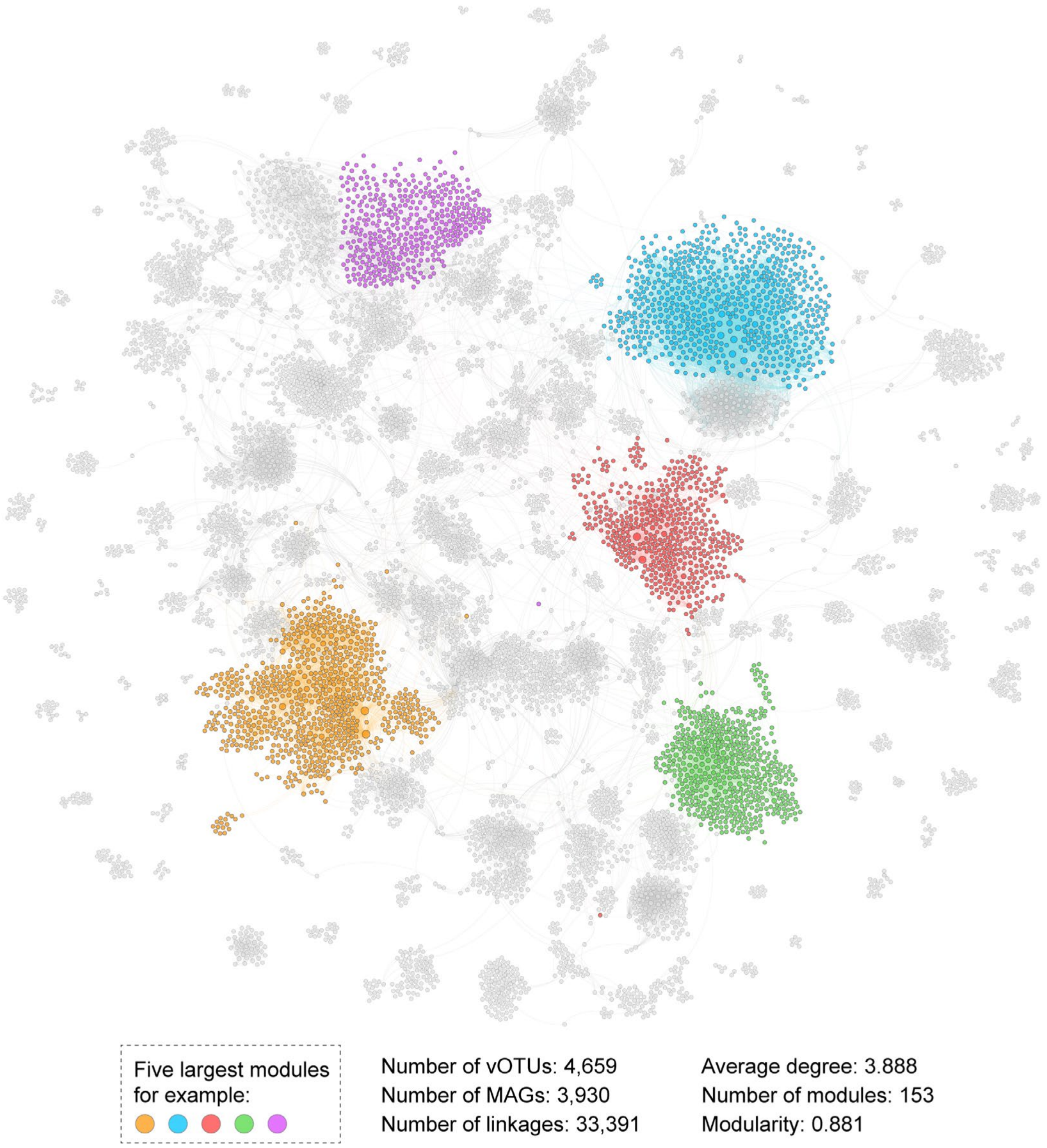

Fig. S16 Co-occurrence network of virus–host interactions. Nodes with or without black outlines represent MAGs or vOTUs, respectively. Each edge marks a specific virus–host linkage. The modularity of the network is calculated using community detection algorithm built in Gephi. Top five modules are shown in different colors. Source data are provided in the Source Data file.

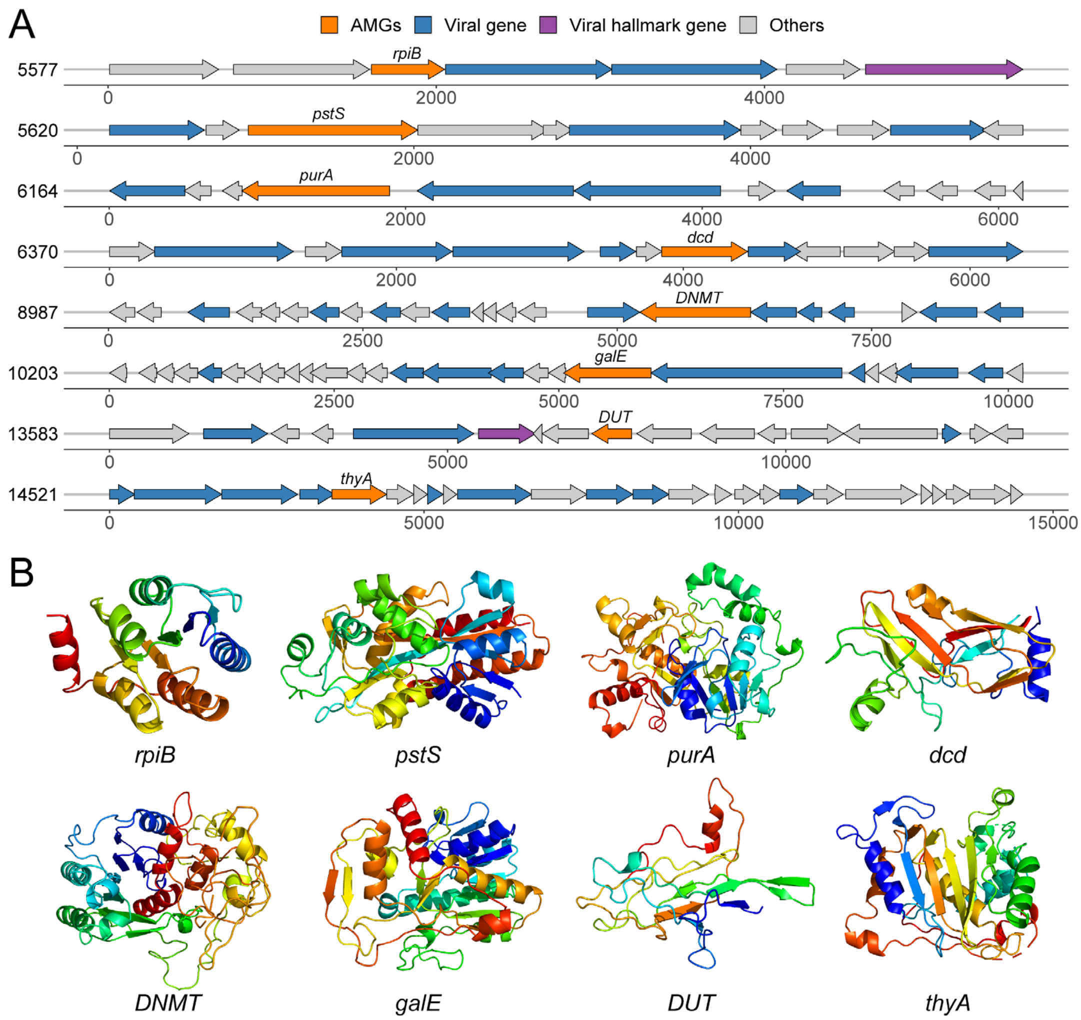

Fig. S17 Genomic context and protein structure of selected virus-encoded AMGs. A Genome map of representative AMG-encoding viral contigs. Each contig is marked by its genome length. **B** Tertiary structures of selected AMGs based on structural modelling using Phyre2. *rpiB*: ribose 5-phosphate isomerase B; *pstS*: phosphate transport system substrate-binding protein; *purA*: adenylosuccinate synthase; *dcd*: dCTP deaminase; *DNMT*: DNA (cytosine-5)-methyltransferase 1; *galE*: UDP-glucose 4-epimerase; *DUT*: dUTP pyrophosphatase; *thyA*: thymidylate synthase. Source data are provided in the Source Data file.

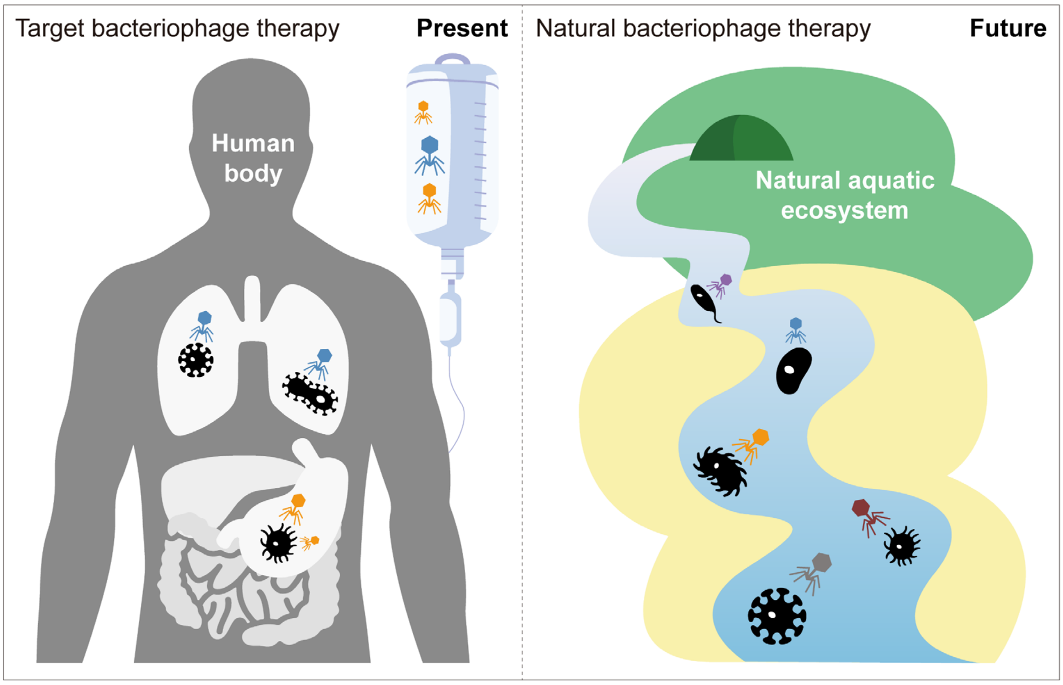

Fig. S18 Outlook of practical utility of natural bacteriophage therapy in natural aquatic ecosystems compared to the target bacteriophage therapy used in human body. Targeted infections by specific viruses have enabled precise treatments of pathogen-induced human diseases in clinical practice. Future studies would be expected to widen the application prospects of natural bacteriophage therapy to eliminate the waterborne pathogens.

Supplemental Tables

Table S1. Sequencing data quantity and region classification for each of the 64 samples in autumn and spring.

Table S2. The number and average length of vOTUs within different quality levels in autumn and spring.

Table S3. Permutational multivariate analysis of variance (PERMANOVA) for statistical significances of viral and bacterial communities spatiotemporally.

Table S4. Annual average TP concentrations (mg/L) measured in the MR-SNWDC (present study) and those observed in other representative river/lake ecosystems (from the Global Freshwater Quality Database). The records are sorted based on the average TP concentration in all years.

Table S5. Virus–host linkages and taxonomic classification of viruses and hosts.

Table S6. Summary of virus-encoded auxiliary metabolism genes (AMGs) identified in the MR-SNWDC.

Supplemental References

1. Bastian M, Heymann S, Jacomy M. Gephi: an open source software for exploring and manipulating networks. ICWSM. 2009;8:361-362.
2. Blondel VD, Guillaume J-L, Lambiotte R, Lefebvre E. Fast unfolding of communities in large networks. J Stat Mech-Theory E. 2008;2008:P10008.
3. Chen W, Wang J, Chen X, Meng Z, Xu R, Duoji D, et al. Soil microbial network complexity predicts ecosystem function along elevation gradients on the Tibetan Plateau. Soil Biol Biochem. 2022;172:108766.
4. Liu B, Arlotti D, Huyghebaert B, Tebbe CC. Disentangling the impact of

contrasting agricultural management practices on soil microbial communities – Importance of rare bacterial community members. Soil Biol Biochem. 2022;166:108573.

5. Nayfach S, Camargo AP, Schulz F, Eloe-Fadrosh E, Roux S, Kyrpides NC. CheckV assesses the quality and completeness of metagenome-assembled viral genomes. Nat Biotechnol. 2021;39:578-585.
6. Hyatt D, Chen GL, LoCascio PF, Land ML, Larimer FW, Hauser LJ. Prodigal: prokaryotic gene recognition and translation initiation site identification. BMC Bioinformatics. 2010;11:119.
7. Huerta-Cepas J, Szklarczyk D, Heller D, Hernández-Plaza A, Forslund SK, Cook H, et al. eggNOG 5.0: a hierarchical, functionally and phylogenetically annotated orthology resource based on 5090 organisms and 2502 viruses. Nucleic Acids Res. 2019;47:D309-D314.
8. Huerta-Cepas J, Forslund K, Coelho LP, Szklarczyk D, Jensen LJ, von Mering C, et al. Fast genome-wide functional annotation through orthology assignment by eggNOG-mapper. Mol Biol Evol. 2017;34:2115-2122.
9. Kieft K, Zhou ZC, Anantharaman K. VIBRANT: automated recovery, annotation and curation of microbial viruses, and evaluation of viral community function from genomic sequences. Microbiome. 2020;8:90.
10. Guo J, Bolduc B, Zayed AA, Varsani A, Dominguez-Huerta G, Delmont TO, et al. VirSorter2: a multi-classifier, expert-guided approach to detect diverse DNA and RNA viruses. Microbiome. 2021;9:37.
11. Shaffer M, Borton MA, McGivern BB, Zayed AA, La Rosa Sabina L, Solden LM, et al. DRAM for distilling microbial metabolism to automate the curation of microbiome function. Nucleic Acids Res. 2020;48:8883-8900.
12. Pratama AA, Bolduc B, Zayed AA, Zhong ZP, Guo JR, Vik DR, et al. Expanding standards in viromics: *in silico* evaluation of dsDNA viral genome identification, classification, and auxiliary metabolic gene curation. PeerJ. 2021;9:e11447.

13. Kelley LA, Mezulis S, Yates CM, Wass MN, Sternberg MJE. The Phyre2 web portal for protein modeling, prediction and analysis. Nat Protoc. 2015;10:845-858.
14. Sigrist CJA, de Castro E, Cerutti L, Cuche BA, Hulo N, Bridge A, et al. New and continuing developments at PROSITE. Nucleic Acids Res. 2012;41:D344-D347.
15. Roux S, Páez-Espino D, Chen I-MA, Palaniappan K, Ratner A, Chu K, et al. IMG/VR v3: an integrated ecological and evolutionary framework for interrogating genomes of uncultivated viruses. Nucleic Acids Res. 2020;49:D764-D775.
16. Jang HB, Bolduc B, Zablocki O, Kuhn JH, Roux S, Adriaenssens EM, et al. Taxonomic assignment of uncultivated prokaryotic virus genomes is enabled by gene-sharing networks. Nat Biotechnol. 2019;37:632-639.
17. Buchfink B, Xie C, Huson DH. Fast and sensitive protein alignment using DIAMOND. Nat Methods. 2015;12:59-60.
18. Nepusz T, Yu HY, Paccanaro A. Detecting overlapping protein complexes in protein-protein interaction networks. Nat Methods. 2012;9:471-472.
